# Wheat TaSPL13‐2B Improves Floret Fertility and Enhances Grain Number per Spikelet Through Jasmonic Acid Signalling Pathway

**DOI:** 10.1111/pbi.70463

**Published:** 2025-11-19

**Authors:** Li Li, Fu Shi, Yaqiong Wang, Yanbin Guan, Ya'nan Wu, Ling Chen, Junli Chang, Mingjie Chen, Jun Xiao, Guangxiao Yang, Yuesheng Wang, Guangyuan He, Yin Li

**Affiliations:** ^1^ The Genetic Engineering International Cooperation Base of Chinese Ministry of Science and Technology, The Key Laboratory of Molecular Biophysics of Chinese Ministry of Education, College of Life Science and Technology Huazhong University of Science & Technology Wuhan China; ^2^ Institute of Food Crops Hubei Academy of Agricultural Sciences Wuhan China; ^3^ Hubei Key Laboratory of Food Crop Germplasm and Genetic Improvement Wuhan China; ^4^ Key Laboratory of Crop Molecular Breeding Ministry of Agriculture and Rural Affairs Wuhan China; ^5^ Key Laboratory of Plant Cell and Chromosome Engineering, Institute of Genetics and Developmental Biology Chinese Academy of Sciences Beijing China; ^6^ University of Chinese Academy of Sciences Beijing China; ^7^ CAS‐JIC Centre of Excellence for Plant and Microbial Science (CEPAMS) Institute of Genetics and Developmental Biology, Chinese Academy of Sciences Beijing China

**Keywords:** floret fertility, jasmonic acid signalling, *TaMADS1*, *TaSPL13‐2B*, wheat

## Abstract

Floret fertility is a key determinant of grain number per spike and an important factor in cereal crop yield. However, the mechanisms by which phytohormone signalling and transcription factors coordinately regulate floret fertility and spikelet development are not well understood, especially in wheat. In this study, we identified the role of jasmonic acid (JA) in the regulation of floret fertility in wheat. TaSPL13‐2B, a JA‐responsive regulator, directly represses the gene expression of the key JA signalling factor *TaJAZ1* to improve floret fertility and increase the number of florets and grains per spikelet. The TaSPL13‐2B‐regulated JA signalling module (TaJAZ1–TaMYC2) contributes to floret fertility by inducing the expression of *TaMADS*1, an E‐class gene critical for floral organ identity and floret meristem activity, and increasing the content of jasmonoyl‐isoleucine (JA‐Ile) by upregulating the expression of genes involved in JA biosynthesis. We further demonstrated that TaSPL13‐2B is a potential target for yield improvement through field trials. Our work provides mechanistic insights into floret fertility and demonstrates that improving floret fertility could be a promising strategy to increase yield.

## 1 Introduction

Wheat ( *Triticum aestivum* L.) is an important staple crop for the global population and a major source of carbohydrates and calories in the human diet (Levy and Feldman 2022). Increasing wheat yield has always been among the major goals of wheat farmers and plant breeders. Wheat yield is a multifactorial trait that is determined mainly by the number of spikes per unit area, the number of grains per spike and the thousand‐grain weight, and the combination of these parameters is used to demonstrate yield potential (Cao et al. 2020; Song et al. 2023). The number of grains per spike is determined by both the number of spikelets per spike (SPS) and the number of grains per spikelet (GPS). Both the number of SPS and the number of GPS are strongly affected by the developmental processes of wheat inflorescence, and the number of GPS is also affected by the fertility of each floret in wheat (Sakuma and Schnurbusch 2020).

During wheat inflorescence development, the spikelet meristem produces 8–12 floret primordia, but only 3–5 primordia survive and form fertile flowers (Sakuma and Schnurbusch 2020; Backhaus et al. 2023). Thus, floret fertility is a determining factor for the number of GPS. Improving floret fertility could be a promising strategy for increasing wheat yield. Floret development comprises two key phases: initiation and death. The maximum number of floret primordia per spikelet peaks during the early reproductive stage until the green anthers stage (GrA), after which only a few florets become fertile by the anthesis stage (AN) (Guo et al. 2016). Floret fertility has been shown to be more strongly associated with the number of surviving florets rather than the number of florets initially produced. Within a spike, central spikelets usually produce the greatest number of grains, whereas most apical and basal spikelets produce no or only a few grains (Bonnett 1966). Most basal spikelets develop first but tend to be smaller, whereas apical spikelets develop last with less time for primordium formation (Backhaus et al. 2022). The variation in floret and grain numbers among the different spikelets suggests differences in floret fertility, although the underlying mechanisms remain largely unknown.

In wheat, floret fertility has been associated with endogenous phytohormone levels (Sun et al. 2024). In cereal crops, the levels of endogenous phytohormones, particularly cytokinins (CKs), auxins and gibberellic acid (GA), affect the survival rate of florets and grain yield (Gallavotti et al. 2004; Yamaguchi et al. 2014; Galli et al. 2015; Boden 2016; Zheng et al. 2016; Youssef et al. 2017; Chen et al. 2021; Tu et al. 2022). For example, the reduced expression of *Cytokinin oxidase/dehydrogenase 2* (*OsCKX2*) increases CK levels and the number of reproductive organs in rice (Ashikari et al. 2005). Additionally, spraying plants with synthetic CK 6‐benzylaminopurine (6‐BA) increases the number of fertile florets and grains per spike in wheat (Zheng et al. 2016). Auxin signalling via genes such as maize *BARREN STALK 1* and its rice ortholog *LAX PANICLE 1* controls reproductive meristem formation (Gallavotti et al. 2004). In barley, auxin and CK concentrations show opposite trends in the apical spikelets and the basal spikelets, suggesting possible coordination between auxin and CK signalling in meristem specification (Boden 2016). GA promotes cell elongation and differentiation by interacting with DELLA proteins and activates *APETALA1* expression by interacting with *SQUAMOSA* promoter binding protein‐like 9 (SPL9) to enforce FM identity (Yamaguchi et al. 2014).

Compared with the roles of auxins, CK and GA in wheat spike development, the role of jasmonic acid (JA) is poorly understood, despite extensive research regarding the effects of JA on flower development and fertility in *Arabidopsis*, tomato, rice, maize and sorghum (Huang et al. 2017, 2023). In sorghum, a TCP transcription factor (TF) regulates panicle development, possibly through JA signalling (Jiao et al. 2018). JA signalling involves three key components: the receptor CORONATINE‐INSENSITIVE 1 (COI1), jasmonate ZIM‐domain (JAZ) repressors and MYC transcriptional activators. Upon the detection of JA‐Ile, COI1 interacts with JAZ repressors to promote the ubiquitination‐mediated degradation of JAZs, releasing MYC TFs to control downstream JA‐responsive genes (Chini et al. 2007; Liu et al. 2021; Song et al. 2021). In *Arabidopsis*, the interaction between MYC2 and ETHYLENE INSENSITIVE3 (EIN3) modulates the antagonism between jasmonate and ethylene signalling pathways (Song et al. 2014). Several MYCs redundantly regulate stamen development and seed production (Qi et al. 2015). In rice, *extra glume 1* (*EG1*) is involved in JA biosynthesis, whereas EG2 represses JA signalling by inhibiting the OsMYC2‐mediated activation of *OsMADS1*, thereby influencing spikelet development (Cai et al. 2014). Although the JAZ–MYC network is well established in *Arabidopsis* and rice, its role in floret fertility in wheat remains unclear.

In addition to phytohormones, TFs play key roles in regulating grass inflorescence architecture (Zhang and Yuan 2014). Forward genetic analyses, genome‐wide association studies (GWASs) (Guo et al. 2017), quantitative trait loci (QTLs) analyses (Saini et al. 2022) and multiomics analyses (Ai et al. 2024; Lin et al. 2024) have revealed that numerous TFs function in the early stages of spike development. These inflorescence regulators are enriched mainly in certain TF families, such as APETALA2/ethylene‐responsive factors (AP2/ERF), MADS‐box and SPL (Li, Zhong, et al. 2018; Chen and Gallavotti 2021; Du et al. 2022; Luo et al. 2023). For example, the MADS‐box TF *PANCILE PHYTOMER2* (*TaPAP2*) inhibits spikelet meristem (SM) initiation and reduces spikelet number (Wang et al. 2017), and the *VRS1* homologue in barley, *GNI‐A1*, affects floret fertility (Sakuma et al. 2019). Among the abovementioned TF families, SPL plays an important role in wheat spike development (Liu et al. 2017; Li et al. 2020; Gupta et al. 2023; Pei et al. 2023). TaSPL3 and TaSPL17 interact with TaDWARF53 to influence spike development (Liu et al. 2017). TaSPL7 and TaSPL15 regulate tiller number, spike length and spikelet number in wheat (Pei et al. 2023). At the transcriptomic level, *TaSPL13* regulates spikelet development (Li et al. 2020), and mutations in the miRNA156 recognition elements (MREs) of TaSPL13 improve multiple traits related to spike architecture and yield (Gupta et al. 2023). Considering the multifaceted roles of SPLs in wheat spike development, further investigations of the detailed functions of TaSPLs and the underlying mechanisms are valuable. In particular, little is known about how these TFs coordinate with phytohormone signalling to control spikelet development in wheat.

Here, we initially measured the concentrations of several phytohormones from basal to apical spikelets and focused on the effects of JA on wheat floret fertility and grain yield. The genetic and biochemical mechanisms responsible for JA‐regulated floret fertility are poorly understood. We hypothesized that JA‐responsive TFs that control inflorescence architecture traits may also be involved in JA‐regulated floret fertility. By utilising gene expression analysis and reverse genetics, we demonstrated that *TaSPL13‐2B* is responsive to JA and strengthens the JA signalling module TaJAZ1–TaMYC2 by repressing the expression of *TaJAZ1*. Subsequently, TaMYC2 activates the expressions of *TaMADS1*, *TaOPR12* and *TaJAR1*, thereby improving floral fertility and promoting JA biosynthesis. The results of field experiments supported *TaSPL13‐2B* as a potential target gene for wheat yield, as its transgenic expression increased the grain yield per unit by 11.7%. Our results suggest that *TaSPL13‐2B* is a key gene that regulates JA signalling during spikelet development and improves floret fertility. More importantly, our study conceptually proves that improving floret fertility could be a promising breeding strategy for wheat yield without an obvious trade‐off between yield component traits.

## 2 Results

### 2.1 JA Increases Grain Number per Spike in Wheat

**FIGURE 1:**
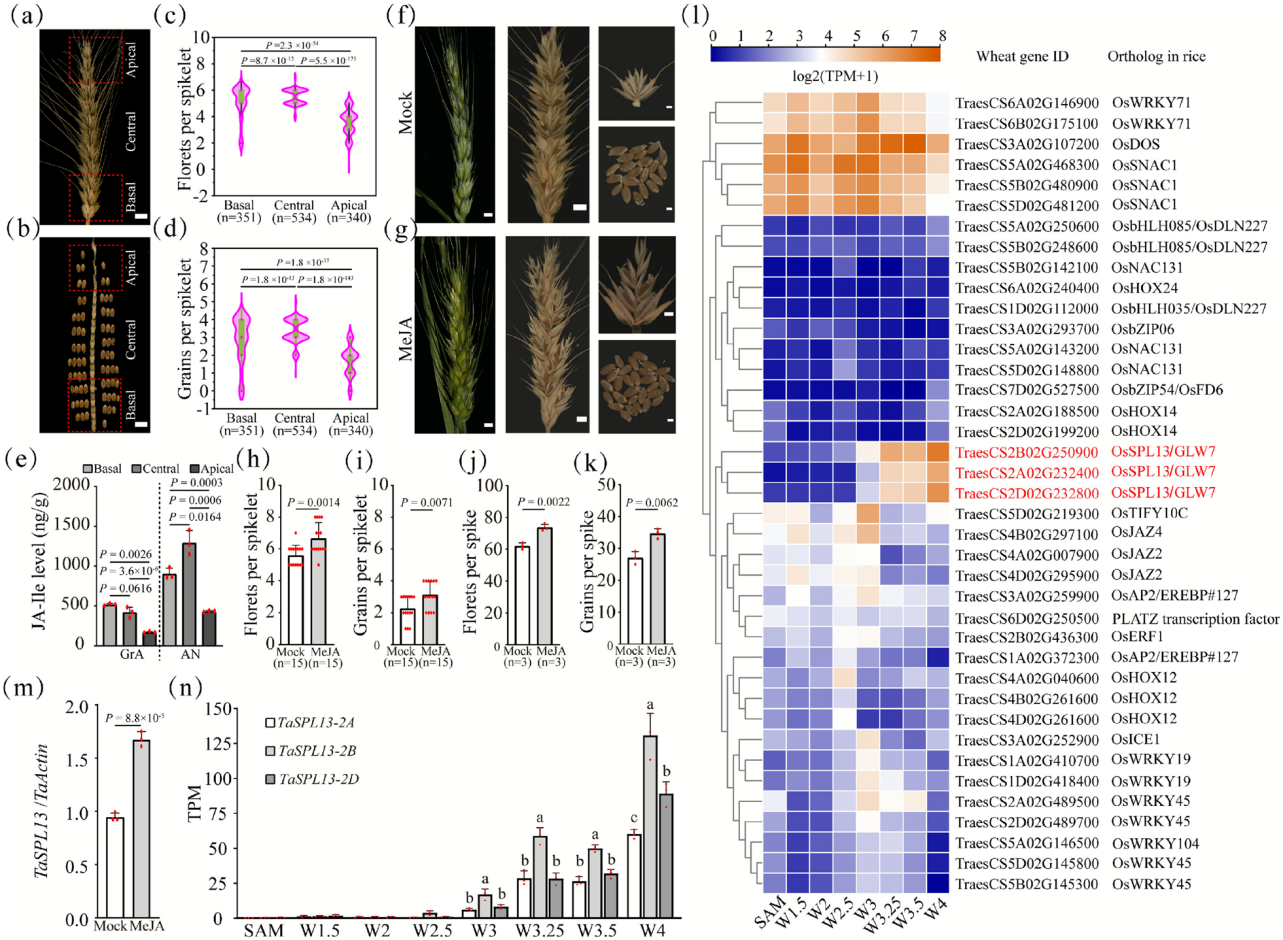
JA increases the number of florets and grains in wheat spikelets. (a) A representative spike of the bread wheat cultivar Bobwhite. Scale bar: 2 cm. (b) The number of grains per spikelet dissected from the spike of *cv*. Bobwhite. Scale bar: 2 cm. (c, d) Statistical analysis of the number of florets and grains between the apical, central and basal spikelets, respectively. (e) Measurement of endogenous JA‐Ile levels in apical, central and basal spikelets. GrA: Green anther stage; AN: Anthesis stage. *n* = 3 biological replicates. (f, g) Representative spike morphology at the floret development and maturity stages and corresponding spikelets and grains per spike from control (f) and MeJA‐treated plants (g). Scale bars: 5 cm for the spike and 1 cm for the spikelets and grains. (h–k) Statistical analysis of the number of florets per spikelet (h), grains per spikelet (i), florets per spike (j) and grains per spike (k) between the control and MeJA treatment groups. Mock indicates the control group. (l) Heatmap showing TFs expression dynamics across spike developmental stages (Lin et al. 2024). The blue colour indicates low expression, and the orange colour indicates high expression. (m) *TaSPL13* expression levels in the spikelets of MeJA‐treated and control plants. (n) Expression levels of *TaSPL13‐2A*, *TaSPL13‐2B* and *TaSPL13‐2D* among different stages of young spikes based on Lin's data (Lin et al. 2024). Differences in gene expression between the *TaSPL13* triads for a given stage were calculated by one‐way variance analysis (ANOVA) and are indicated with different letters (*p* < 0.05). For the remaining figures, significant differences were determined by two‐tailed Student's *t*‐test. Dots show the data distribution. The number of samples and exact *p* values are shown in the figures.

To gain insights into the differences in floret fertility between apical and basal spikelets, we first measured the number of florets and grains per spikelet at three spikelet positions (i.e., the apical, central and basal spikelets) in the wheat *cv*. Bobwhite under field conditions (Figure 1a,b). The basal and central spikelets produced greater numbers of florets and grains per spikelet than did the apical spikelet (Figure 1c,d), confirming the differences in floret fertility between these spikelets. Immature spikelets from the apical, central and basal spikelets were sampled at the GrA and AN stages to determine their phytohormone contents (i.e., IAA, GA, CK and JA). JA‐Ile content was significantly greater in the basal and central spikelets than in the apical spikelets and correlated well with the number of fertile florets and grains of the corresponding spikelet (Figure 1e). The IAA and CK (cZ) concentrations at the three positions were seemingly correlated with the number of florets and grains per spikelet at the GrA stage but not at the AN stage (Figure S1a,b). We did not observe any correlations between GA metabolite abundance and floret fertility among the spikelets (Figure S1c–f). The molecular mechanisms underlying auxin‐ or CK‐mediated control of inflorescence architecture have been previously described (Galli et al. 2015; Zheng et al. 2016; Tu et al. 2022). Surprisingly, our data support the important role of JA in determining wheat floret fertility because the fold changes in the JA‐Ile content among the three spikelet positions correlated well with the corresponding floret fertility, and the absolute levels of JA‐Ile were the greatest among the measured phytohormones. To verify the effects of JA on floret fertility, wheat plants were treated with 1 mM Methyl Jasmonate (MeJA) from the tillering stage through the beginning of grain development. Compared with the control plants, the MeJA‐treated plants presented significantly greater numbers of florets and grains per spikelet (Figure 1f–k), indicating that JA is indeed involved in regulating the number of grains per spikelet.

Spike development and meristem differentiation are orchestrated by phytohormone signalling and master transcriptional regulators (Zhang and Yuan 2014). Floret fertility is determined during the late stages of wheat spike development. Thus, we sought to identify transcriptional regulators involved in the JA‐mediated regulation of floret fertility in wheat using reverse genetics. Transcriptional regulators are likely JA responsive and might be among the TF families known to conservatively control inflorescence development in other grasses (Li, Zhong, et al. 2018; Chen and Gallavotti 2021; Du et al. 2022; Luo et al. 2023). We integrated wheat transcriptome datasets from JA‐treated spikes and from spikes at different developmental stages, leading to the identification of 488 JA‐responsive differentially expressed genes (DEGs) (Figure S1g; Table S1). The Gene Ontology (GO) enrichment analysis revealed that these DEGs are involved in various biological functions, including the response to jasmonic acid, root meristem growth and stamen development (Table S2) (Feng et al. 2017; Li, Fu, et al. 2018; Qi et al. 2019; Lin et al. 2024). Among the DEGs, 39 genes encode members of multiple TF families (e.g., HOX, bHLH, AP2 and SPL) that function in grass inflorescence architecture (Figure 1l; Table S1). Since the currently available RNA‐seq datasets were collected at early stages of wheat spike development (Lin et al. 2024), we intentionally pinpointed JA‐responsive TFs with a gradual increase in expression at the W4 stage and afterward. Notably, the expression of all three *TaSPL13* homoeologs (*TaSPL13‐2A* (TraesCS2A02G232400), *TaSPL13‐2B* (TraesCS2B02G250900) and *TaSPL13‐2D* (TraesCS2D02G232800)) increased in young developing spikes (Figure 1l). We also confirmed that *TaSPL13* is JA‐responsive, with increased expression levels in MeJA‐treated Bobwhite spikelets (Figure 1m). During spike development, compared with its homoeologs *TaSPL13‐2A* and *TaSPL13‐2D*, *TaSPL13‐2B* had higher expression levels (Figure 1n). The differences in the promoter sequences could be associated with the expression levels between the *TaSPL13* homoeologs, whereas the high similarities in the coding regions among the *TaSPL13* triads and in the encoded protein sequences suggest that the TaSPL13 homoeologous proteins may be functionally conserved (Figures S2–S4).

Previously, MREs of three *TaSPL13* homoeologs were edited using CRISPR/Cas9, leading to an approximately 2‐fold increase in *TaSPL13* expression with pleiotropic effects on plant height, flowering time, tiller number and grain size and number (Gupta et al. 2023). Additionally, we verified that *TaSPL13* regulates spikelet development (Li et al. 2020). Despite these reported lines of genetic evidence for TaSPL13, the molecular mechanism through which TaSPL13 regulates spike development and how TaSPL13 is associated with the JA‐mediated control of floret fertility remains unknown. Overall, we demonstrated that TaSPL13 could be a candidate regulator involved in JA‐mediated regulation of floret fertility.

### 2.2 TaSPL13‐2B Increases Grain Number per Spikelet in Wheat

**FIGURE 2:**
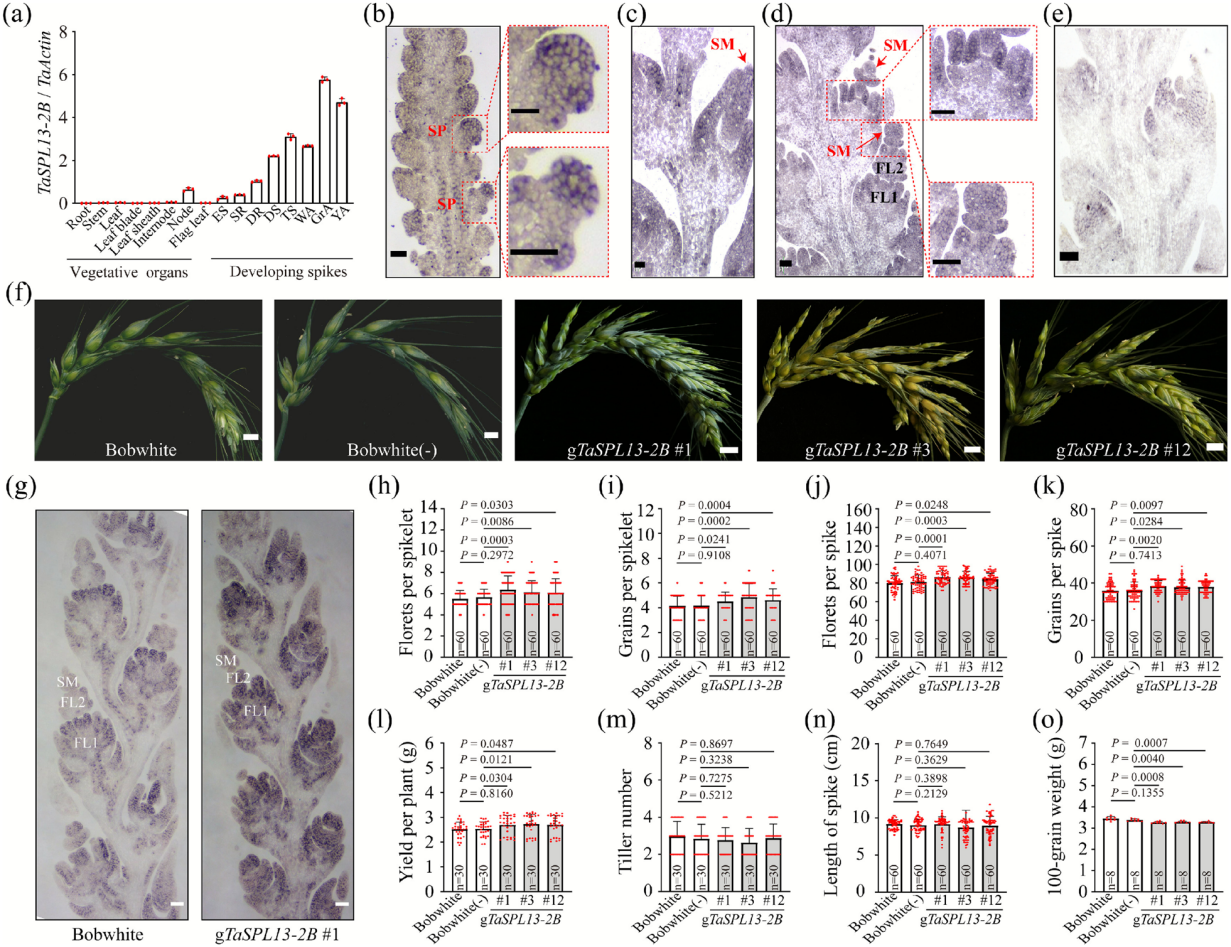
*TaSPL13‐2B* increases the number of florets per spike and grains per spike. (a) Transcription profiling of *TaSPL13‐2B*. ES: Elongation stage; SR: Single ridge stage; DR: Double ridge stage; DS: Differentiation stage; TS: Terminal spikelet stage; WA: White anther stage; GrA: Green anther stage; YA: Yellow anther stage. Dots show the data distribution. (b–e) RNA ISH showing *TaSPL13‐2B* spatial expression patterns during wheat spike development in the DR (b), DS (c) and TS (d) stages using antisense probes. Negative control with a sense probe in the TS stage (e). SP: Spikelet primordium; SM: Spikelet meristem; FL: Floret. Scale bars: 100 μm. (f) Comparison of spike morphology between the g*TaSPL13‐2B* transgenic and control lines. Scale bar: 1 cm. (g) RNA ISH results for *TaSPL13‐2B* in wild type and transgenic wheat spikes in the TS stage of development. Scale bars: 100 μm. (h–o) Statistical analysis of yield‐related parameters, including the number of florets per spikelet (h), number of grains per spikelet (i), number of florets per spike (j), number of grains per spike (k), yield per plant (l), tiller number (m), length of spike (n) and hundred‐grain weight (o), between the transgenic and control lines. Statistical differences were calculated using a two‐tailed Student's *t*‐test. The four *p* values represent the significant differences between the null‐segregant line and each of the three transgenic lines relative to the wild type plants, and their exact *p* values are indicated in the figure. Dots show the data distribution; n indicates the sample number.

To investigate the molecular mechanism by which TaSPL13 regulates wheat spikelet development, we first examined the spatiotemporal expression patterns of *TaSPL13‐2B* across spike developmental stages. Quantitative real‐time PCR (qRT‐PCR) analysis revealed that *TaSPL13* was barely expressed in vegetative stage tissues and that the expression levels increased in the immature spikes. The expression levels peaked during the GrA and yellow anther stages (Figure 2a). *TaSPL13‐2B* mRNA was localised using in situ hybridization (ISH) at the double ridge stage (DR), differentiation stage (DS) and terminal spikelet stage (TS) of wheat spikes. *TaSPL13‐2B* was detected in the spikelet primordium (SP) (Figure 2b) and SM (Figure 2c). At the TS stage, *TaSPL13‐2B* mRNA was present in the SM and floret primordia (Figure 2d).

Previously, we reported *TaSPL13‐2B* transgenic lines of wheat with an altered spikelet development phenotype, laying a foundation for pinpointing the molecular mechanism of TaSPL13‐2B (Figure 2f) (Li et al. 2020). The genomic DNA fragment harbouring the *TaSPL13‐2B* coding region and ~2.2 kb 5′ upstream region was used to construct and generate the transgenic wheat lines. Notably, the transgenic *TaSPL13‐2B* lines, which contained higher *TaSPL13‐2B* expression levels as a result of the transgenic addition of the 2B copy with the highest abundance among the three *TaSPL13s*, were driven by its native promoter (Figure 2g). This strategy helped clarify the biological functions of SPL in spike development and avoid irrelevant phenotypes that were potentially caused by a strong and constitutive expression pattern. Three independent nonsegregant lines were obtained (namely, g*TaSPL13‐2B* #1, g*TaSPL13‐2B* #3 and g*TaSPL13‐2B* #12; Figure S5a). At different stages of spike development, the expression levels of *TaSPL13‐2B* in the transgenic lines were significantly greater than those in the wild‐type plants (Figure S5b). Our previous work demonstrated that *TaSPL13‐2B* regulates spike development, but its effect on grain yield has not been explicitly evaluated (Li et al. 2020). The T_7_ generation of *TaSPL13‐2B* transgenic and control plants, including wild‐type and the transgenic null‐segregant line, was used for phenotype confirmation and statistical analysis of yield‐related traits.

In the greenhouse, compared with the control plants, all three transgenic lines produced significantly greater numbers of florets per spikelet (Figure 2h), grains per spikelet (Figure 2i), florets per spike (Figure 2j) and grains per spike (Figure 2k). Compared with that of the null‐segregant line, the average grain yield per plant increased by 7.9% (Figure 2l). There are no significant differences in tiller number or spike length (Figure 2m,n). However, the average hundred‐grain weight decreased by 3.0% compared with that of the null‐segregant line (Figure 2o; Table S3). Our results demonstrated that TaSPL13‐2B regulates wheat spikelet development and increases the number of fertile florets per spikelet.

### 2.3 TaSPL13‐2B Participates in the JA Signalling Pathway and Increases Floret Fertility

**FIGURE 3:**
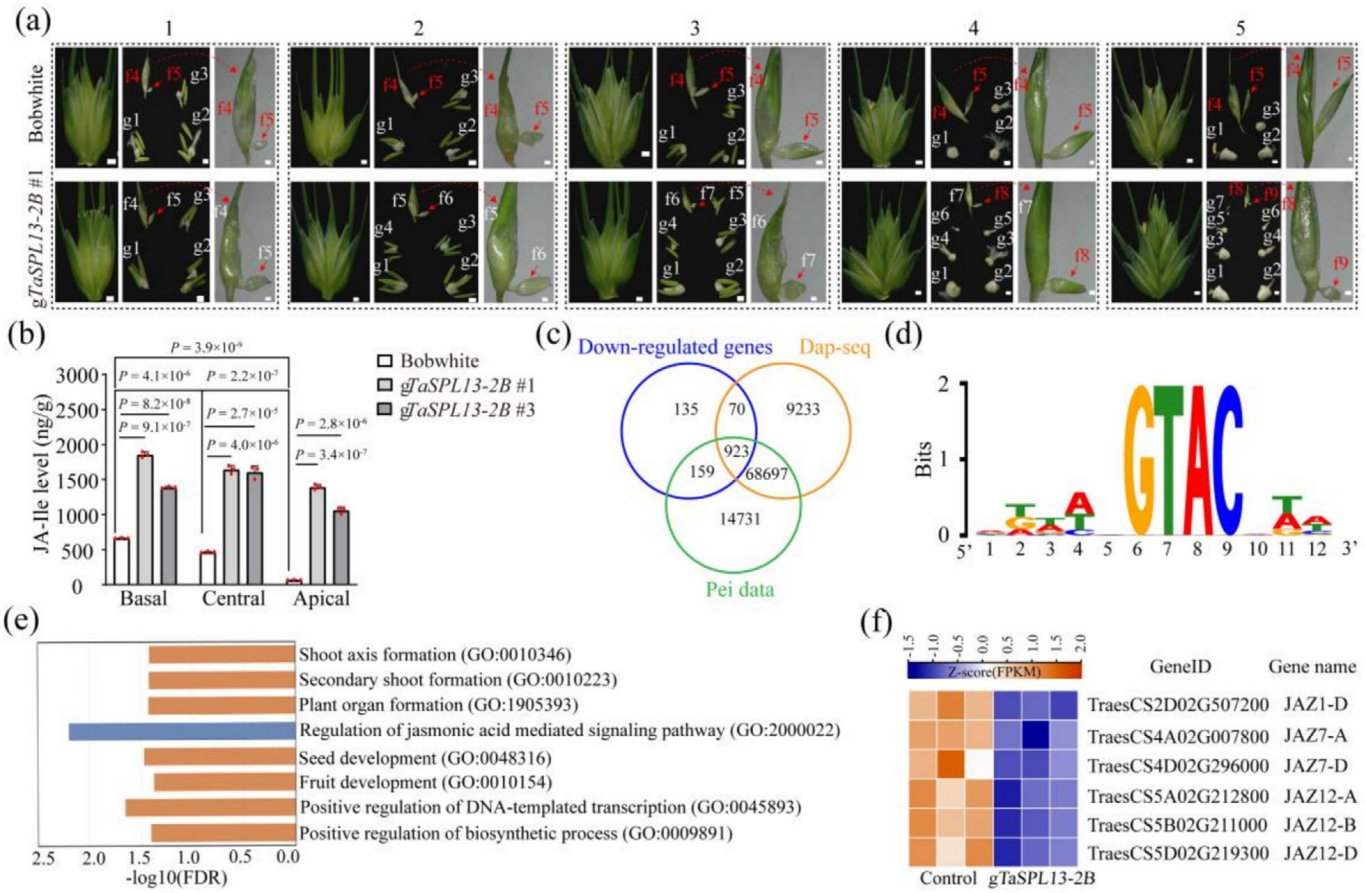
*TaSPL13‐2B* participates in the JA signalling pathway to enhance floret fertility. (a) Comparison of florets from the central spikelet between the wild type and the *TaSPL13‐2B* transgenic line (g*TaSPL13‐2B* #1) at different developmental time points. 1–5 indicate consecutive time points of the spikelet, each of which includes the morphology and the anatomical view of the spikelet and the morphology of the apical‐most floret. “f” represents florets, with fertile florets labelled in white font and sterile florets labelled in red font. “g” represents grains. Scale bars: 100 μm. (b) The content of JA‐Ile in the upper florets of basal, central and apical spikelets from two *TaSPL13‐2B* transgenic lines and the wild type, with the differences in JA‐Ile content determined by two‐tailed Student's *t*‐test. (c) Venn diagram showing overlapping genes between *TaSPL13‐2B* binding targets revealed by our DAP‐seq data (orange circle), Pei's DAP‐seq data (green circle) (Pei et al. 2023), and the downregulated genes (down‐DEGs, blue circle) between *TaSPL13‐2B* transgenic and wild type plants determined by RNA‐seq. (d) The putative DNA binding motif of *TaSPL13‐2B* identified by DAP‐seq is based on the most frequent motif in the promoter of the target gene. (e) GO enrichment analysis of the TaSPL13‐2B target genes, identified by DAP‐seq and RNA‐seq, highlights the involvement of JA signalling in the *TaSPL13‐2B* transgenic lines. (f) Heatmap showing the expression patterns (in Z‐score of FPKM) of several JA‐signalling genes identified by DAP‐seq and RNA‐seq differentially expression analyses. Orange indicates high expression, while blue indicates low expression.

To further elucidate the specific spikelet developmental process in which TaSPL13 is involved, we compared spikelet development between the transgenic wheat lines and the wild type plants across multiple stages, ranging from the elongation stage (ES) to the early stages of kernel development (Figure 3a and Figure S6a). The microscopy and scanning electron microscopy (SEM) results revealed that compared with the wild type plants, the *TaSPL13‐2B* transgenic line did not seemingly alter the spike developmental process until the GrA stage, as the number of florets was similar (Figure S6a,b). Notably, the apical florets from the *TaSPL13‐2B* transgenic lines were stable and able to continue floret development, whereas those from the wild type plants failed to develop into floral organs at the floret development stage (Figure S6c). Compared with the wild type plants, the *TaSPL13‐2B* transgenic lines presented greater numbers of fertile florets and grains (Figure 3a). To further verify whether TaSPL13‐2B regulates spikelet development through the JA signalling pathway, we measured the JA‐Ile content in the upper florets of spikelets at the apical, central and basal positions of the wild type plants and the two transgenic lines. Compared with that in the wild type plants, the JA‐Ile content in the *TaSPL13‐2B* transgenic lines dramatically increased across all three positions, with central and basal spikelets showing higher JA‐Ile levels than apical spikelets (Figure 3b). Moreover, in the transgenic lines, the *TaSPL13‐2B* expression levels at all three positions were significantly greater than those in the wild type plants (Figure S6d). JA‐responsive marker genes were also significantly upregulated in the *TaSPL13‐2B* transgenic lines (Lorenzo et al. 2004; Leon‐Reyes et al. 2010; An et al. 2022) (Figure S7). Taken together, these results prompted us to hypothesize that *TaSPL13‐2B* may directly regulate JA metabolism and/or signalling to increase floret fertility and GPS in wheat.

**FIGURE 4:**
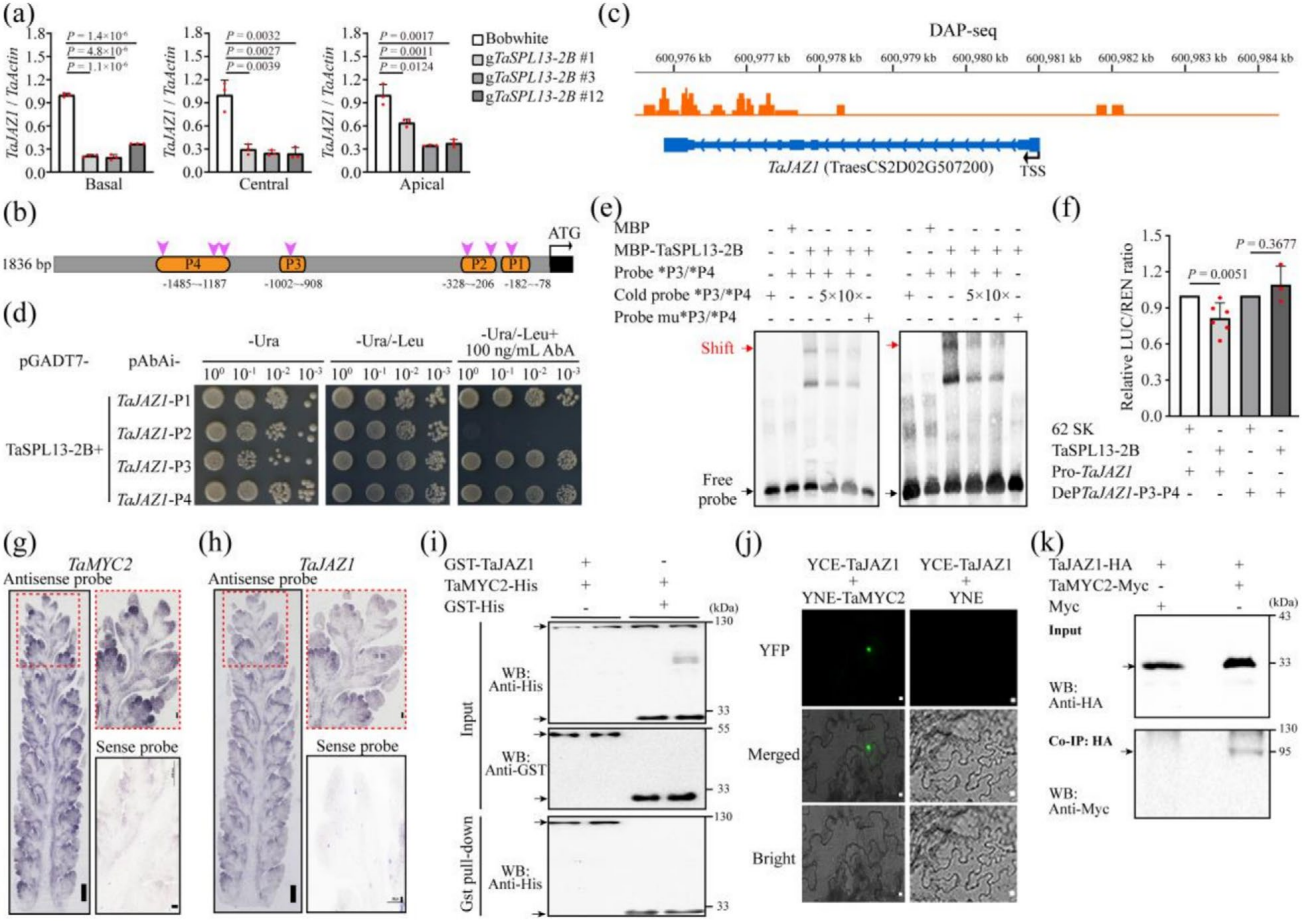
Direct binding of TaSPL13‐2B to the *TaJAZ1* promoter region repress *TaJAZ1* expression and release of TaMYC2. (a) Quantitative RT–PCR analysis of *TaJAZ1* gene expression in the upper florets of spikelets at the basal, central and apical positions in the wild type and three transgenic lines. (b) Diagram of the *TaJAZ1* promoter region; the pink triangles indicate putative TaSPL13‐2B binding motifs. The orange‐highlighted regions (P1‐P4) indicate the promoter fragments used for Y1H. (c) Integrative Genomics Viewer image of the *TaJAZ1* gene in the DAP‐seq reads. The arrow indicates the direction of the gene. (d) Y1H assessment of the interaction between TaSPL13‐2B and the *TaJAZ1* promoter fragment. (e) EMSA of TaSPL13‐2B binding to *TaJAZ1* promoter fragments P3 (left) and P4 (right). The arrows indicate protein–DNA complexes and free probes, respectively. * indicates the ~50 bp fragments of P3 and P4 used in EMSA. (f) Luciferase activity in tobacco leaves transiently expressing the TaSPL13‐2B and Pro‐*TaJAZ1* reporter genes. DePTaJAZ1‐P3‐P4 indicates that, on the basis of the results of the EMSA, the binding sites for TaSPL13‐2B were deleted in the promoter region of the *TaJAZ1* gene. The LUC/REN ratio indicates the relative activity of the promoter. (g, h) RNA ISH detected *TaMYC2* (g) and *TaJAZ1* (h). Expression at the TS stage using antisense probes. Sense probes for *TaMYC2* and *TaJAZ1* served as controls. Scale bars: 100 μm. (j, k) A series of biochemical experiments, including GST pull‐down (i), BiFC (j) and Co‐IP (k), confirmed the interaction between the TaJAZ1 and TaMYC2 proteins. Scale bar: 100 μm in (j). The arrowheads indicate the corresponding MYC2 and JAZ1 protein bands. The data are the means ± SDs. Significant differences were determined using a two‐tailed Student's *t*‐test. Dots show the data distribution, with the exact *p* value for each comparison provided in figures (a) and (f).

To explore the molecular network through which *TaSPL13‐2B* regulates JA‐Ile content, signalling and floret fertility, we performed RNA‐seq and DNA‐affinity purification sequencing (DAP‐seq) to elucidate TaSPL13‐2B‐regulated gene expression and identify the direct binding targets of TaSPL13‐2B. Additionally, comparisons between our TaSPL13‐2B DAP‐seq data and the previous results revealed substantial consistency (Pei et al. 2023), identifying a total of 69 620 in vitro targets of TaSPL13‐2B (Figure 3c) enriched with a conserved GTAC binding motif of the SPL family (Figure 3d; Table S4). The electrophoretic mobility shift assay (EMSA) further confirmed that TaSPL13‐2B binds to the GTAC motif (Figure S8a). Moreover, a series of multiomics analyses supported our hypothesis that TaSPL13‐2B is directly involved in the JA signalling pathway. First, GO enrichment analysis of the TaSPL13‐2B targets revealed enrichment of the term “response to JA” (GO: 0009753; Figure S8b). Second, terms related to inflorescence development, regulation of meristem development and jasmonic acid hydrolase were enriched in the DEGs between the *TaSPL13‐2B* transgenic lines and the wild type plants (Figure S9a). A small number of previously identified genes involved in the regulation of spike and meristem development were enriched among the upregulated DEGs. In contrast, a significantly larger set of genes associated with spike and inflorescence development were enriched among the downregulated DEGs (Figure S9b). Additionally, TaSPL13‐2B functions as a transcriptional repressor (Li et al. 2020; Pei et al. 2023), and notably, the functional term “regulation of the JA‐mediated signaling pathway” was significantly enriched in the downregulated DEGs (Figure S9c). Third, we integrated the TaSPL13‐2B‐bound targets and TaSPL13‐2B‐downregulated genes in the spikelets and identified a total of 923 high‐confidence TaSPL13‐2B target genes. Among these targets, developmental‐ and JA‐related functions were significantly enriched (e.g., “plant organ formation” and “regulation of JA mediated signaling pathway”) (Figure 3e; Table S5). Moreover, several *JAZ* family members (e.g., *JAZ1‐D*, *JAZ7‐A*/*D* and *JAZ12‐A/B/D*) were significantly downregulated in the *TaSPL13‐2B* transgenic lines (Figure 3f). In developing young spikes, the *TaJAZ1‐A/D* was exhibited the predominant expression among the *JAZ* family members (Table S6). Quantitative RT–PCR analysis revealed lower *TaJAZ1* expression in the basal, central and apical spikelets of the *TaSPL13‐2B* transgenic lines than in those of the wild type plants (Figure 4a). We also identified *TaJAZ1*, the wheat ortholog of the rice JA signalling repressor OsJAZ1/EG2, as an important target of TaSPL13‐2B (Cai et al. 2014; Ye et al. 2024). We focused on the regulation between TaSPL13‐2B and *TaJAZ1*, considering TaJAZ1 as one of the major JAZ proteins involved in wheat spikelet development.

### 2.4 TaSPL13‐2B Is Involved in the TaJAZ1‐TaMYC2 Module to Regulate JA Signalling

We sought to confirm whether TaSPL13‐2B controls JA signalling through direct repression of *TaJAZ1*. Several binding motifs for TaSPL13‐2B were found in the *TaJAZ1* promoter (Figure 4b), consistent with the identification of *TaJAZ1* as a target gene in the DAP‐seq assay (Figure 4c). Yeast one‐hybrid (Y1H) assays and EMSAs revealed that TaSPL13‐2B bound to two regions of the *TaJAZ1* promoter (designated P3 and P4) (Figure 4d,e). Dual luciferase assays further demonstrated that TaSPL13‐2B significantly inhibited luciferase expression driven by the *TaJAZ1* promoter, whereas TaSPL13‐2B failed to repress luciferase expression when a P3‐ and P4‐deleted *TaJAZ1* promoter was used (Figure 4f).

JAZ proteins are typically involved in the JA signalling pathway through interactions with the F‐box protein COI1 and several JA‐responsive TFs (Thines et al. 2007; Yan et al. 2007). JAZ proteins bind to COI1 in the SCF^CoI1^ complex, resulting in the ubiquitination and subsequent degradation of JAZs (Xu et al. 2002). To further determine the role of TaJAZ1 in the JA response, yeast two‐hybrid (Y2H) assays were conducted to test the interactions between TaJAZ1 and the TaCOI proteins, which are homologous to OsCOI1a, OsCOI1b and OsCOI2 (Nguyen et al. 2022; Wang et al. 2023). The results revealed that the TaJAZ1–TaCOI3‐4A interaction was dependent on coronatine (COR), a JA‐Ile mimic, but not on MeJA or dimethyl sulfoxide (DMSO). No interactions were detected between TaJAZ1 and other TaCOI proteins (Figure S10a). The JAZ‐COI1 interaction mediates the degradation of JAZ proteins via the 26S proteasome (Liu et al. 2021). To determine whether TaJAZ1 degradation could also be mediated by the 26S proteasome in our case, we transiently expressed *TaJAZ1‐LUC* in *Nicotiana benthamiana* leaves and treated them with 100 μM MeJA or the 26S proteasome‐specific inhibitor MG‐132. After 1 h of MeJA treatment, the luminescence signal was markedly reduced. However, when MG‐132 was administered, this decrease in the luminescence signal was clearly inhibited (Figure S10b). Immunoblotting analysis of total protein using an anti‐HA antibody further confirmed that MeJA significantly decreased TaJAZ1 protein levels, which were attenuated by MG‐132 (Figure S10c). These results indicated that TaJAZ1 could be degraded by the 26S proteasome.

Among the JAZ‐targeted TFs, MYC2 is a well‐studied master regulator of JA signalling (Kazan and Manners 2013). We examined the spatiotemporal expression patterns of *TaMYC2* and *TaJAZ1* to determine whether TaMYC2 could act as a major partner of TaJAZ1 during spikelet development. *TaMYC2* and *TaJAZ1* were highly expressed in young spikes (Figure S11a,b). *ISH* results revealed that *TaMYC2* and *TaJAZ1* were strongly expressed in the FM at the TS stage (Figure 4g,h). The spatiotemporal coexpression of *TaSPL13*, *TaMYC2* and *TaJAZ1* suggests that TaMYC2 and TaJAZ1 may work together in JA signalling during floret fertility formation. Our Y2H results indicated that TaJAZ1 and TaMYC2 directly interact through the Jas domain of TaJAZ1 and the N‐terminus of TaMYC2 (Figure S11c,d). Subcellular localization analysis revealed that TaMYC2 was located in the nucleus, whereas TaJAZ1 was located in the nucleus and cytoplasm (Figure S12a,b). Glutathione S‐transferase (GST) pull‐down, coimmunoprecipitation (Co‐IP) and bimolecular fluorescence complementation (BiFC) assays were performed. The GST‐TaJAZ1 protein was effectively pulled down by TaMYC2‐His (Figure 4i). The BiFC results demonstrated that TaJAZ1 and TaMYC2 interact in the nucleus, and our Co‐IP data further confirmed this interaction (Figure 4j,k). Taken together, these results indicated that TaJAZ1 directly interacts with TaMYC2 in vitro and in vivo during spikelet development.

### 2.5 TaMYC2 Activates TaMADS1 and JA Biosynthesis

**FIGURE 5:**
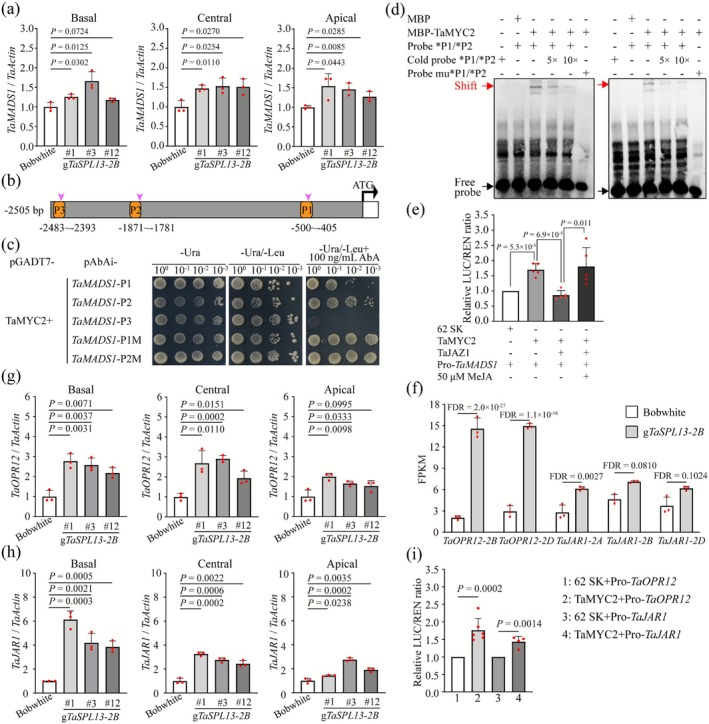
TaMYC2 affects the expression of the downstream transcription factor *TaMADS1* and the JA biosynthetic genes *TaOPR12* and *TaJAR1*. (a) *TaMADS1* expression in the upper florets of spikelets at the basal, central and apical positions in the wild type and three transgenic lines. (b) Schematic of the *TaMADS1* promoter region, with pink triangles indicating putative TaMYC2 binding motifs. The orange‐highlighted regions (P1–P3) indicate the promoter fragments used for Y1H. (c) Y1H analysis of the interaction between TaMYC2 and *TaMADS1* promoter fragments. (d) EMSA results of TaMYC2 binding to *TaMADS1* promoter fragments P1 (left) and P2 (right). The arrows indicate protein–DNA complexes and free probes, respectively. * indicates the ~50 bp fragments of P1 and P2 used in EMSA. (e) Luciferase activity in tobacco leaves transiently expressing the TaMYC2 protein and Pro‐*TaMADS1*. TaMYC2 and TaJAZ1 indicate that these genes were injected simultaneously into tobacco leaves, and TaMYC2 and TaJAZ1 were simultaneously injected into tobacco leaves, followed by 50 μM MeJA. (f) RNA‐seq data showing differences in the expression of the *TaOPR12* and *TaJAR1* genes, with FDR values indicating significance. (g, h) Expression levels of *TaOPR12* (g) and *TaJAR1* (h) in the upper florets of spikelets at the basal, central and apical positions in the wild‐type and three transgenic lines. (i) Luciferase activity in tobacco leaves transiently expressing the TaMYC2 protein and the *TaOPR12* or *TaJAR1* reporter gene. The LUC/REN ratio indicates the relative activity of the promoter. Exact *p* values determined by the two‐tailed Student's *t‐*test are shown in the Figures (a) and (e–i).

**FIGURE 6:**
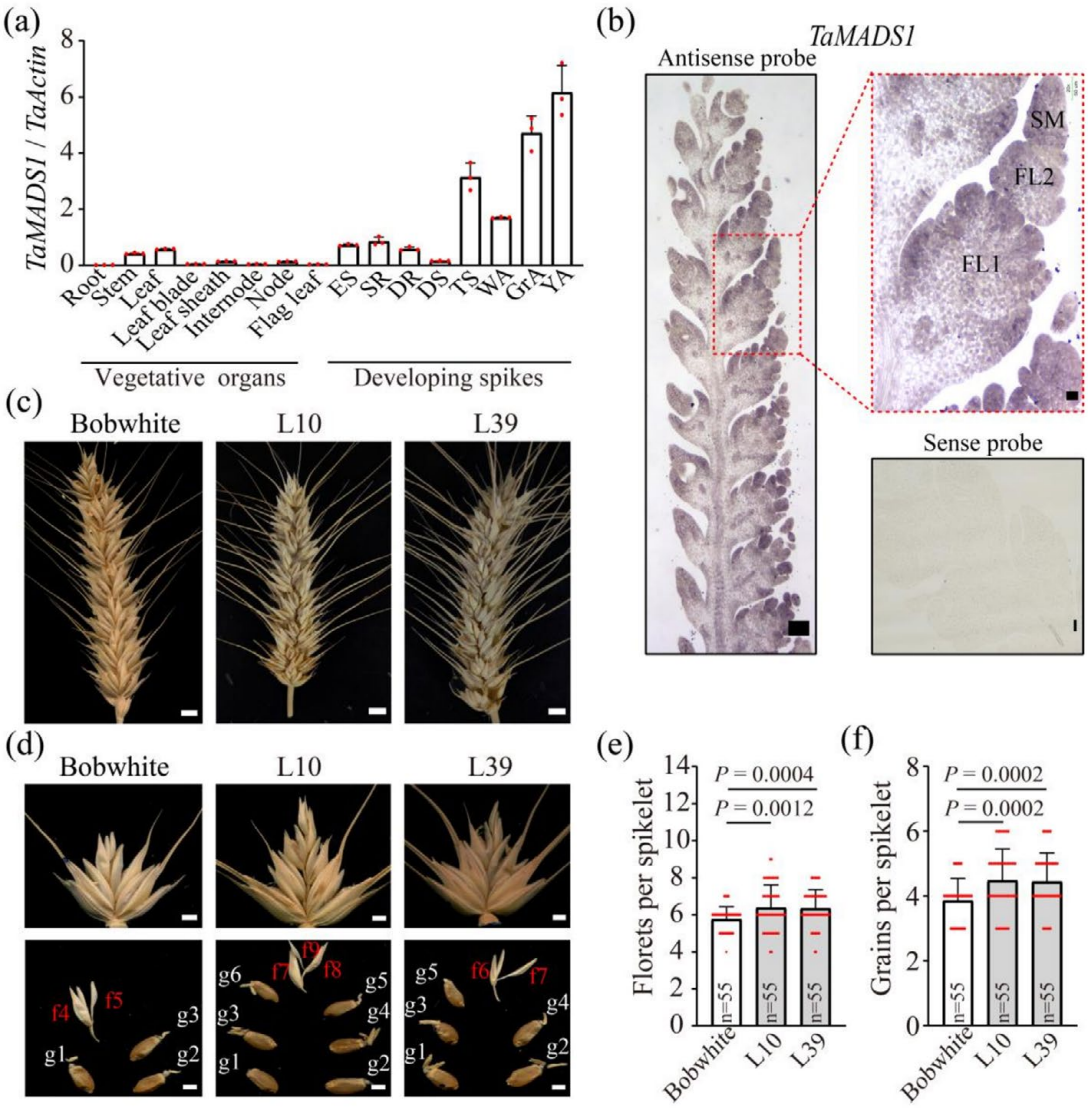
The number of grains per spikelet was increased in the *TaMADS1* transgenic wheat line. (a) Transcriptional profiles of *TaMADS1*. (b) RNA ISH detected *TaMADS1* expression at the TS stage using antisense probes. *TaMADS1* sense probes as controls. (c) Differences in the morphology of wheat spikes between two *TaMADS‐* transgenic lines (L10 and L39) and the wild‐type plants during the seed maturation stage. Scale bars: 0.5 cm. (d) Representative spikelets and grains from two *TaMADS1‐*transgenic lines and the wild‐type plants. f, Florets, sterile florets are marked in red. g, Grains. Scale bars: 0.3 cm. (e–f) The number of florets and grains per spikelet between the two *TaMADS1* transgenic lines and the wild‐type, with dots indicating the data distribution. Statistical differences were calculated with two‐tailed Student's *t*‐test with the *p* values provided in the figures.

MADS‐box TFs are the major regulators of floral organ development (Callens et al. 2018). A group of E‐class MADS‐box TFs in the ABCDE model is critical for floral organ determinacy and spikelet development (Cui et al. 2010). In light of this, we identified MADS‐box genes that were differentially expressed between the *TaSPL13‐2B* transgenic lines and the wild type, whose orthologs in model species are functionally important for floral identity and development. Indeed, the expression of the ortholog of an E‐class *OsMADS1* (a.k.a., *TaSEP1‐A3‐2*, designated *TaMADS1* hereafter) was upregulated in the *TaSPL13‐2B* transgenic wheat, whereas the expression of other *TaMADS* genes was downregulated (Figure S13). Expression analysis revealed that *TaMADS1* expression was significantly upregulated in the *TaSPL13‐2B* transgenic lines across the basal, central and apical spikelets compared with that in the wild type (Figure 5a). Moreover, HvMADS1 directly regulates the cytokinin‐degrading enzyme *HvCKX3* to integrate temperature and cytokinin homeostasis, thereby maintaining spike morphology in barley (Li et al. 2021). OsMADS1 plays a crucial role in regulating the differentiation pattern of the floral primordium and determining the flower meristem (Chen et al. 2006). These data from grass species imply that *TaMADS1* might be one of the major TFs downstream of JA signalling, contributing to improved floret fertility in the *TaSPL13‐2B* transgenic lines. Several lines of evidence support these findings. First, we validated the transactivation ability of TaMYC2 (Figure S14). Second, *TaMADS1* was upregulated at several stages of spike development (from the TS stage to the YA stage), and ISH indicated that *TaMADS1* is expressed in the floret and spikelet meristem (Figure 6a,b), demonstrating that *TaJAZ1*, *TaMYC2* and *TaMADS1* are coexpressed in floral meristematic cells. Third, Y1H, EMSA and dual luciferase assays confirmed that TaMYC2 activated the expression of *TaMADS1* (Figure 5b–e). Fourth, we tested whether TaJAZ1 inhibits the TaMYC2‐mediated activation of *TaMADS1*. When TaJAZ1 and TaMYC2 were cotransformed into tobacco leaves, TaJAZ1 repressed TaMYC2‐mediated *TaMADS1* expression to the control level; however, the addition of MeJA mitigated the TaJAZ1‐mediated repression on *TaMADS1* expression (Figure 5e). These biochemical experiments support a classic JA signalling mechanism in wheat in which upon sensing the JA signal, JA‐Ile binds to the JAZ‐COI1 coreceptor complex, resulting in the degradation of JAZ (Chini et al. 2007; Thines et al. 2007; Yan et al. 2009). These results suggest that *TaMADS1* is a major TF that acts downstream of the TaJAZ1–TaMYC2 JA signalling module to maintain floret fertility in wheat spikelets.

In the *TaSPL13‐2B* transgenic spikelets, the endogenous JA‐Ile level was significantly greater than that in the wild type (Figure 3b), suggesting that TaSPL13‐2B or the downstream TaJAZ1–TaMYC2 module may regulate JA biosynthesis during floret development. Recent studies have shown that MYC2 directly binds to the promoters of several genes involved in JA biosynthesis (e.g., *LOXs*, *AOS*, *JAR1* and *OPR3*) and increases their expression levels in *Arabidopsis* (Hou et al. 2010; Van Moerkercke et al. 2019; Zander et al. 2020). RNA‐seq data revealed that the expression levels of *TaOPR12* and *TaJAR1* were significantly increased in *TaSPL13‐2B* transgenic spikelets (Figure 5f). Moreover, the qRT–PCR results confirmed that these two genes were significantly upregulated in the basal, central and apical spikelet tissues of the *TaSPL13‐2B* transgenic lines compared with those of the wild type (Figure 5g,h). Dual luciferase assays confirmed that TaMYC2 binds to the promoters and activates the expression of *TaOPR12* and *TaJAR1*, thereby increasing the JA content in the floral meristem (Figure 5i).

### 2.6 TaMADS1 Positively Regulates Grain Number per Spikelet

To further substantiate TaMADS1 as a major TF contributing to floral identity and fertility, the coding sequence of *TaMADS1* was inserted into the pAHC25 plasmid driven by the maize ubiquitin promoter and transformed into the wheat *cv*. Bobwhite using the biolistic bombardment method. Two transgenic lines were obtained (*TaMADS1*‐L10 and *TaMADS1*‐L39). Compared with the wild‐type plants, the *TaMADS1* transgenic plants produced significantly higher numbers of florets and grains per spikelet (6.37 vs. 5.78 florets and 4.47 vs. 3.87 grains per spikelet, respectively; Figure 6c–f). The consistent phenotypes between the *TaMADS1‐* and *TaSPL13‐2B*‐transgenic lines suggest that TaMADS1 probably acts as one of the major TFs downstream of the JA signalling pathway to regulate spikelet development and holds the potential for improving wheat yield.

In rice, OsSPL13 has been demonstrated to regulate panicle development with breeding value, and *OsSPL13* overexpression increases the number of branches and grains per panicle; however, the underlying mechanism has not been investigated (Si et al. 2016). Heterologous expression of *TaSPL13‐2B* in rice also increased the number of branches and grains per panicle (Li et al. 2020). It is unclear whether the SPL‐JA signalling‐mediated regulation of reproductive development is conserved between rice and wheat. Our results suggest that OsSPL13 directly represses *OsJAZ1* expression (Figure S15), suggesting that JA signalling and its regulatory gene modules could be conservatively recruited, whereas the regulatory action and networks might be complex.

### 2.7 TaSPL13‐2B Improves the Grain Yield of Wheat in the Field

**FIGURE 7:**
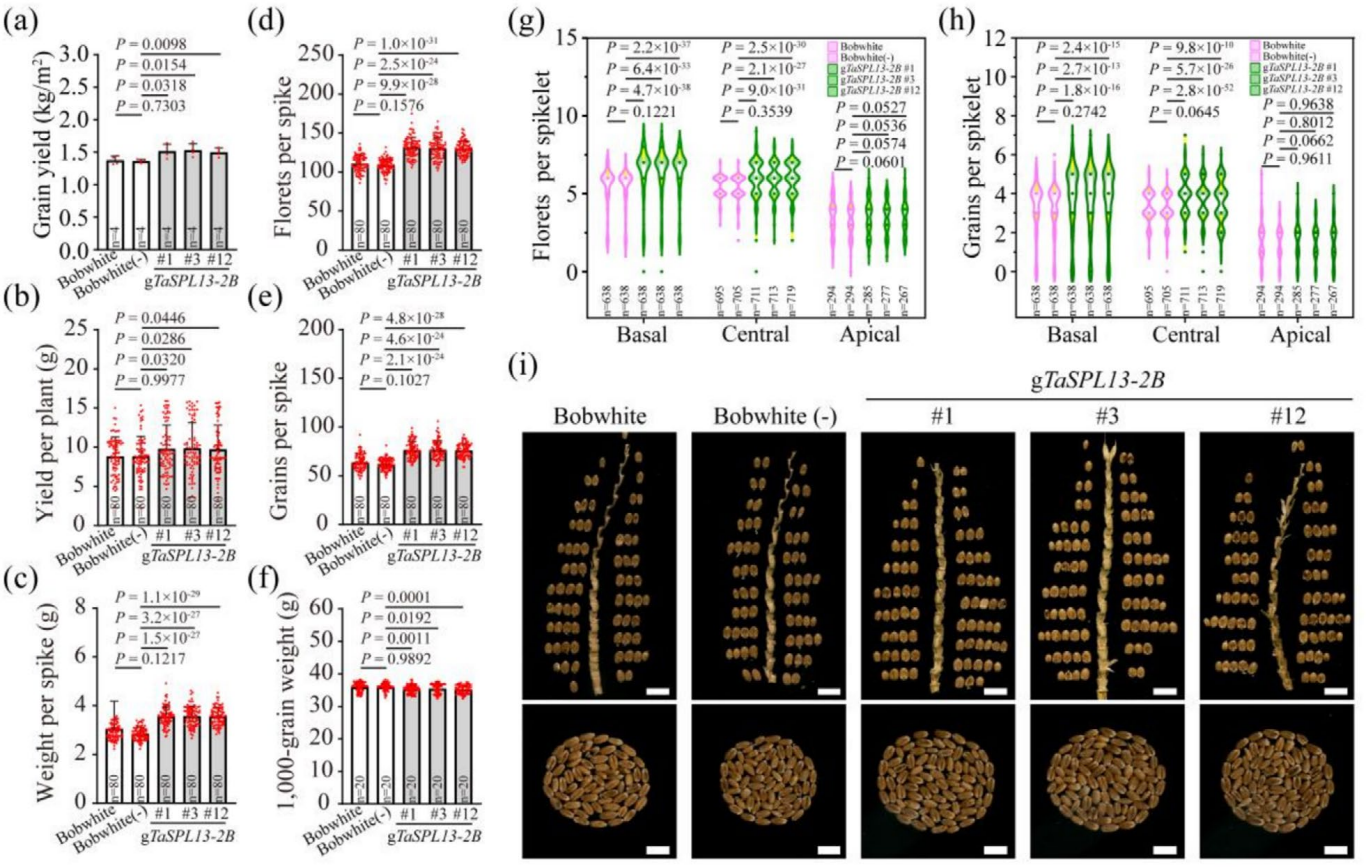
Compared with those of the control lines, the yield traits of the three *TaSPL13‐2B* transgenic lines improved. The experiments were conducted in the 2022/23 field season in the Hongshan experimental field with a completely randomised block design. (a–h) Statistical analysis of several yield‐related traits between the *TaSPL13‐2B* transgenic and control lines in the field experiment, including grain yield (a), yield per plant (b), weight per spike (c), florets per spike (d), grains per spike (e), thousand‐grain weight (f), florets per spikelet (g), and grains per spikelet (h) in the basal, central and apical parts of field‐harvested spikes. Statistical differences were calculated using a two‐tailed Student's *t*‐test. The four *p* values represent the significant differences between the null‐segregant line and each of the three transgenic lines relative to the wild‐type plants, and their exact *p* values are indicated in the figure. Dots show the data distribution; n indicates the sample numbers. (i) Comparisons of grains per spikelet along the spike, and the number of grains per spike between the *TaSPL13‐2B* transgenic and control lines. Scale bars: 0.7 cm.

To assess the utility of TaSPL13‐2B, yield experiments were performed using field designs under multiple environmental conditions. The yield component traits of wheat include grain numbers per spike (GNP), thousand‐grain weight (TGW) and effective tiller number (ETN), which are usually complexly balanced. We systematically evaluated the yield component traits of the *TaSPL13‐2B* transgenic lines under three field environmental conditions. In the 2021‐Hongshan field experiments, compared with the null‐segregant line, the *TaSPL13‐2B* transgenic lines presented significantly greater grain yield per plant (10.9% greater on average) but a slight decrease in TGW (2.1% lower on average) (Figure S16; Table S7). In the 2022/23 field season, the *TaSPL13‐2B* transgenic lines presented significantly greater grain yields per plot, with an average increase of 11.1% and 12.2% in the 2022‐Hongshan and 2022‐Xinzhou fields, respectively (Figure 7, Figures S17 and S18). The significant increase in yield caused by TaSPL13‐2B was largely due to a greater number of florets per spike and number of grains per spike and thus a greater yield per plant, whereas only mild compensation in TGW was associated with transgenic *TaSPL13‐2B* (decreased by 1.9% and 2.5% in the 2022‐Hongshan and 2022‐Xinzhou fields, respectively), and the ETN and spike length did not differ between the transgenic and control lines under all three environmental conditions (Tables S7–S9). To further address whether the grain yield increased because of significantly greater numbers of florets and grains per spikelet, we analysed several hundred spikelets per line in each field experiment. Interestingly, compared with those from the control lines, the numbers of florets and grains per spikelet from the basal and central spikelets of the *TaSPL13‐2B* transgenic lines were greater. For example, in 2022‐Hongshan, an average of 6.4 basal florets versus 5.4 basal florets and 3.8 basal grains versus 3.2 basal grains were recorded between the transgenic and control lines, respectively. The numbers of florets and grains per apical spikelet were similar between the transgenic and control lines (Figure S19). In brief, multienvironment field experiments demonstrated that the *TaSPL13‐2B* transgene could be a useful approach for improving floret fertility and grain number per plant and, thus, grain yield without an apparent trade‐off in terms of other yield component traits.

## 3 Discussion

### 3.1 TaSPL13‐2B Increases Wheat Grain Yield by Regulating Floret Fertility Within Spikelets

Increasing yield is among the main objectives of wheat breeding. The number of grains per spike, which is strongly influenced by floret fertility, is a major determinant of grain yield. Wheat spikelets typically produce up to 12 floret primordia, but more than 70% of the florets are aborted (Sakuma et al. 2019). Theoretically, increasing floret fertility by increasing floret primordia activity and/or promoting floret organ development could be a promising breeding strategy for improving wheat yield.

In this context, the novelty and significance of our work lie in two aspects. First, we elucidated the effects of JA on wheat floret fertility and revealed for the first time that TaSPL13‐2B regulates the core module of JA signalling, TaJAZ1–TaMYC2, to promote JA signalling and metabolism. Second, we demonstrated that genetic manipulation of *TaSPL13* could be an effective approach to improve wheat yield and holds potential for the molecular breeding of wheat. Importantly, our experiments (MeJA‐treated, *TaSPL13‐2B* and *TaMADS1*‐transgenic plants) support the idea that improving floret fertility is a promising strategy for increasing the number of grains per spike, thus improving wheat yield.

Wheat yield component traits (mainly GNP, TGW and ETN) are interconnected. Trade‐off phenomena are often observed when one of these yield traits is manipulated by breeding or biotechnological approaches. In our case, the *TaSPL13*‐*2B* transgenic lines had increased floret fertility, with an average 11.1%–12.2% increase in yield per plot, but the TGW was reduced by only 1.9%–2.5% in Hongshan and Xinzhou (Tables S8 and S9), respectively, indicating the potential of *TaSPL13‐2B* as a promising target gene for yield in wheat breeding. Notably, the increase in grain yield is the balance between the grain number and TGW in this case. Although this characteristic might not be favoured by breeders, the tiling‐deletion screening approach could be applied to overcome gene pleiotropy and trade‐offs.

### 3.2 Spatiotemporal Crosstalk Between Phytohormones Orchestrates Spike Development

In addition, our work demonstrates the importance of JA signalling in reproductive organ development, particularly in the formation of floret fertility and/or the floral meristem, which may be conserved among cereal crops from a developmental biology perspective. Notably, JA signalling‐mediated regulation of spikelets and/or florets is known to occur in other cereal crops and *A. thaliana* . In rice, mutation of the JA biosynthesis‐related gene *eg1* results in defects in spikelet development and altered floral organ number (Cai et al. 2014), whereas mutation of *OsJAR1* decreases the JA concentration and reduces fertility (Xiao et al. 2014). In sorghum, *msd1* regulates pedicellate spikelet development by activating JA biosynthesis (Jiao et al. 2018). These previous reports, together with our work, strongly support the idea that JA metabolism and signalling are required to maintain floral development and fertility across cereal crops, even though the required JA concentration gradients and their modes of action may differ between species. Future work should focus on elucidating the potential coordination between different phytohormones (JA, IAA and CK), which could provide a more in‐depth understanding of the molecular networks that control floret fertility. For example, at the GrA stage of the wheat spike, cZ and IAA levels were higher in the central and basal spikelets than in the apical spikelets, with an apical‐to‐basal hormonal gradient similar to that of JA, suggesting possible coordination or crosstalk between these phytohormones (Figure S1).

We particularly noticed that the IAA and cZ levels were correlated with the floret numbers among the apical, central and basal spikelets at the GrA stage, whereas these correlations were not present at the AN stage. Our hypothetical model indicates that TaSPL13 promotes JA accumulation and signalling, which in turn activates *TaSPL13* (Figures 1m, 3, 4, 5), suggesting a possible feed‐forward loop between TaSPL13 and JA metabolism. It is reasonable to speculate that IAA and/or cZ might act upstream of the SPL13‐JA regulatory module to regulate this potential feed‐forward loop. The phytohormone contents of different parts of the spike have also been reported in barley and wheat (Youssef et al. 2017; Sun et al. 2024). In barley, the auxin concentration was low in the apical parts of the spike but high in the basal parts, whereas CK concentrations showed the opposite trends, suggesting that there was crosstalk between auxin and CK during meristem specification (Boden 2016). Nevertheless, the top‐to‐bottom concentrations of auxin and CK increased significantly in each wheat spikelet. The distribution of the phytohormone gradients between the inflorescences could be explained by the fact that the wheat inflorescence meristem is indeterminate, whereas in barley, it is determinate (Youssef et al. 2017; Sakuma and Schnurbusch 2020). Furthermore, the tissues, sampling stages and methodologies used for hormone measurements varied among the studies (Figure 1) (Youssef et al. 2017; Sun et al. 2024). These studies highlight the necessity of capturing the spatiotemporal dynamics of phytohormones and/or hormonal responses in gene expression as key information to enhance our knowledge of the regulation of inflorescence development. Accordingly, spatial transcriptomics represents a powerful approach for providing high‐resolution maps of phytohormone responses and is necessary for fully understanding spikelet development in the future (Fu et al. 2023; Wang et al. 2024).

### 3.3 Multilayered Negative‐Feedback Mechanisms Function as “Brakes” to Prevent Overactivation of JA Signalling

In this study, we demonstrated a positive feedback pathway of JA signalling; however, continuous activation of JA signalling is detrimental. Thus, negative feedback is required to fine‐tune or repress the possible overactivation of JA signalling. Previous studies have demonstrated that JAZ protein stability and repressor activity are regulated by several mechanisms, including alternative splicing, posttranslational modifications and protein–protein interactions. During rice spikelet development, protein arginine methyltransferase (OsPRMT6a) serves as a JA signalling switch via arginine methylation of OsJAZ1, increasing its interaction with OsCOI1a/OsCOI1b. This interaction leads to the degradation of OsJAZ1 and the release of OsMYC2, thereby activating (or repressing) JA‐repressive genes. In the *osprmt6a‐1* mutant, reduced OsJAZ1 arginine methylation impaired this interaction, stabilised OsJAZ1 and repressed JA responses (Dong et al. 2024). In *Arabidopsis*, alternative splicing of JAZ10 involves the retention of a conserved Jas intron, generating a noncanonical JAZ10 protein with a truncated or lacking Jas motif in the C‐terminus, which affects the interaction strength with COI1 in the presence of JA‐Ile, protein stability and repressive activity (Moreno et al. 2013). The mediator subunit MED25 controls the JA‐induced recruitment of PRP39a and PRP40a to *JAZ6* loci to facilitate the full splicing of the Jas intron, preventing the excessive desensitisation of JA responses (Wu et al. 2020, 2025).

In addition, JA signalling termination occurs via TFs that compete with MYC2. JAMs that are phylogenetically close to MYCs, but lack an activation domain directly bind to the G‐box in the promoters of JA‐responsive genes whose specificity is similar to that of MYCs. Antagonism between JAM repressors and MYC activators affects JA‐mediated transcriptional reprogramming (Sasaki‐Sekimoto et al. 2013; Song et al. 2013). MYC activity levels can also be controlled at the protein level (Song et al. 2025). Regulation of the MYC protein level facilitates the termination of MYC activity and the resetting of the JA signalling pathway. Constitutive photomorphogenic 1 (COP1) is a RING‐finger E3 ubiquitin ligase whose activity decreases the abundance of the MYC protein in the dark (Chico et al. 2014). The BTB/POZ‐MATH (BPM3) protein is a CUL3‐based E3 ubiquitin ligase stabilised by JA that can target and ubiquitinylate MYCs to reset JA signalling, suggesting a negative feedback regulatory mechanism to control the protein abundance and activity of MYCs (Chico et al. 2020). The transcriptional corepressor TOPLESS recruits HDA6 and HDA9 to decrease the histone acetylation around the regulatory regions of MYC2 target genes, thereby inhibiting the expression of JA‐responsive genes (An et al. 2022). In summary, several mechanisms are known to maintain the balance of JA signalling by regulating *JAZ* alternative splicing, JAZ protein stability, MYC protein levels and histone modifications.

TaMYC2 is widely recognised as the master regulator of JA signalling and orchestrates plant growth and development (Kazan and Manners 2013). Recent studies have shown that wheat lines carrying the loss‐of‐function *TaMYC2‐A1* allele exhibit increased spikelet numbers, spikelet densities, spikelet numbers per spike and grain numbers per spike (Lin et al. 2024). These findings are consistent with our observation that *TaSPL13‐2B* expression in transgenic wheat positively regulates grain number, suggesting that TaSPL13‐2B modulates wheat spike development by regulating *TaMYC2* expression. As an activated TF, TaMYC2 not only increases the expression of JA biosynthesis‐related genes but also may promote *TaSPL13‐2B* transcription, thereby amplifying JA signalling during spike development in wheat. Building on these insights, we will now dissect the dynamic TaSPL13‐2B–TaMYC2 interplay across spike development, clarifying how this module exerts positive or negative feedback on the JA signalling pathway, and ultimately reveal its precise role in wheat spike formation.

### 3.4 Revealing the Interactions Between Environmental Factors and the TaSPL13‐2B‐JA Signalling Module in the Regulation of Wheat Floret Fertility

Several aspects merit further study on the basis of the present findings. In the greenhouse, compared with wild type plants, *TaSPL13‐2B* transgenic lines presented an increased number of florets per spikelet, whereas only central and basal spikelets produced more florets under field conditions, suggesting that environmental factors may regulate *TaSPL13‐2B* and/or JA signalling during spikelet development. The novel CRISPR/Cas9‐derived mutations of *TaSPL13* have demonstrated breeding value for improving multiple traits (Gupta et al. 2023). Temperature, photoperiod and nutrient availability have been documented as environmental signals that regulate wheat spikelet formation (Boden et al. 2015; Liu et al. 2023; Zhang et al. 2023). At higher temperatures, the reduction in floret fertility is attributed to a decrease in the soluble sugar content (Liu et al. 2023). The photoperiod‐insensitive gene *Ppd‐1a*, an early‐ flowering gene, reduces the number of fertile florets by decreasing floret survival (Boden et al. 2015). The overall effect of N availability on spike fertility is mediated mainly by the mortality of floret primordia, with a much weaker effect on the maximum number of florets initiated (Zhang et al. 2023). More rigorous studies are needed to determine the environmental factors that affect *TaSPL13‐2B* expression and the underlying genetic basis, which will aid in the understanding of the *TaSPL13‐2B*‐JA signalling pathway and its interactions with environmental factors. In turn, understanding the molecular basis of the interactions between JA signalling and environmental factors on floret fertility would aid in precise yield improvement for a given agroecological condition.

### 3.5 TaSPL13‐2B Regulation and Divergent Functions of E‐Class *TaMADS* Genes

The classical ABCDE model was first established for dicotyledonous plants and has been adopted for grasses, including wheat and rice (Zhang and Yuan 2014). Grasses have evolved a diverse set of E‐class genes that play critical roles in floral organ identity and floral meristem determinacy (Zhang and Yuan 2014; Schilling et al. 2020; Wang et al. 2024). In our study, several E‐class *TaMADS* genes were differentially regulated by TaSPL13‐2B during spike development (Figure S13). Given that *MADS‐box* genes are heavily involved in floral identity and differentiation in cereals and that E‐class *TaMADS* genes are expanded in wheat compared to rice, elucidating the functions of these *TaMADS* genes in the future is important. Additionally, *TaMADS1* is a tandemly duplicated gene located in the wheat selection sweep, whereas the other *TaMADS1* duplicates seem not to be induced by JA (Callens et al. 2018). Exploring the potential functional divergence of *TaMADS1* and its duplicates would be valuable.

**FIGURE 8:**
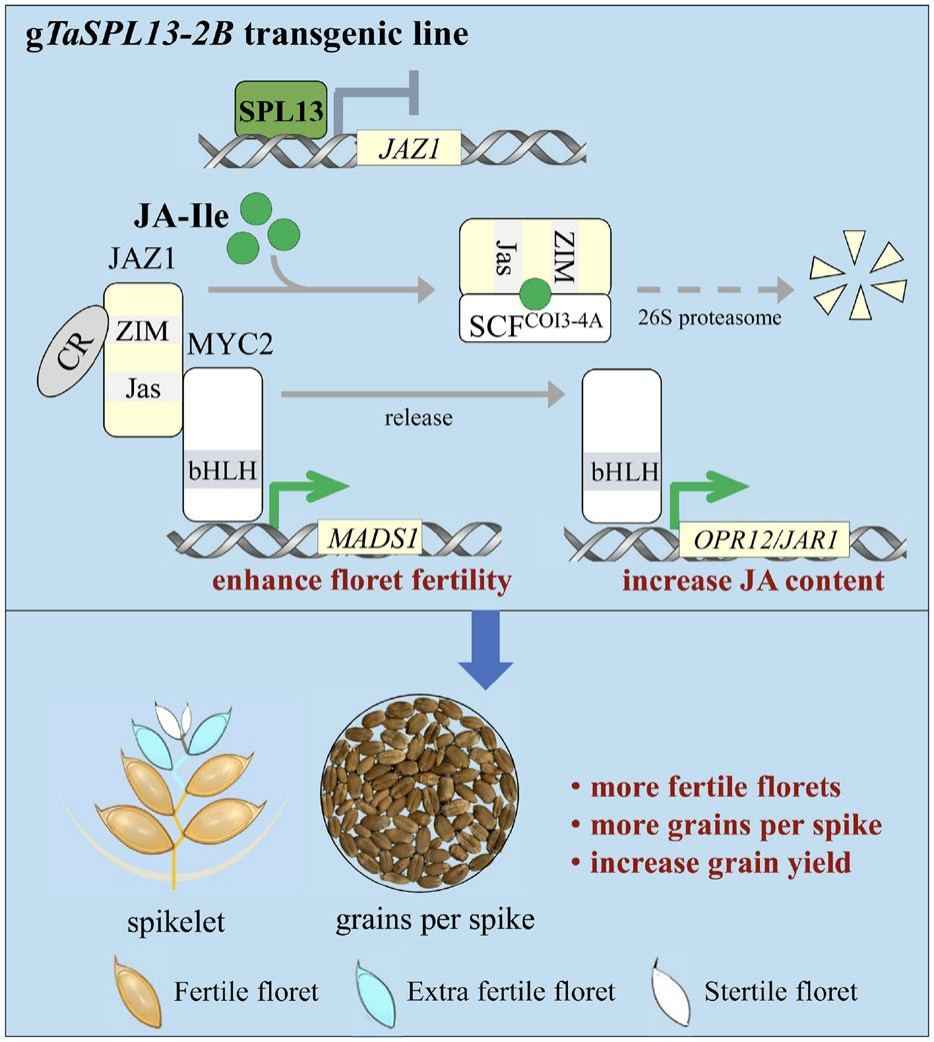
Working model for the TaSPL13‐mediated regulation of the JA signalling module and floret fertility in wheat. In our model, the classical JA signalling component TaJAZ1 interacts with TaMYC2 and represses the TaMYC2‐mediated activation of downstream genes. When the expression of *TaSPL13‐2B* is increased, *TaSPL13‐2B* represses *TaJAZ1* expression by directly binding to the promoter, allowing the release of TaMYC2, a key transcription factor involved in JA signalling. Subsequently, TaMYC2 activates the expression of *TaMADS1* to increase floret fertility and increases the expression of the JA biosynthesis‐related genes *TaOPR12* and *TaJAR1*, likely leading to an increase in the JA content and improving floret fertility. Upon sensing the JA signal, TaJAZ1 proteins are likely ubiquitinated, recruited to interact with the TaCOI3‐4A and SCF^COI3‐4A^ complex, and degraded by the 26S proteasome on the basis of previously established knowledge of the conserved JA signalling pathway. CR, Co‐repressors.

In summary, our work demonstrates the importance of JA in the regulation of spikelet development in wheat. Our data also provide new molecular insights into the transcriptional regulation of JA signalling, which contributes to floret fertility. A model illustrating the ability of transgenic *TaSPL13*‐*2B*‐mediated molecular responses to increase floret fertility and grain yield is shown in Figure 8. In *TaSPL13*‐*2B* transgenic wheat, the repression of TaJAZ1 releases the JA‐responsive TF TaMYC2, allowing the activation of both the floret primordia regulator *TaMADS1* and the JA biosynthetic genes *TaOPR12* and *TaJAR1*. The proof of concept that JA metabolism and/or signalling increases floret fertility and grain yield provides new opportunities for identifying target genes that control floret fertility, such as *TaSPL13*‐*2B* and *TaMADS1*, as presented in our work. These potential target genes hold significant translational value for breeding to increase yield, as they might by pass the trade‐off among yield component traits and may be applicable to other cereal grasses with similar spike architectures.

## 4 Materials and Methods

### 4.1 Plant Materials and Growth Conditions

For endogenous phytohormone assays and *ISH*, RNA‐seq, DAP‐seq and expression analyses, three independent *TaSPL13‐2B* T_7_ transgenic lines and two independent *TaMADS1* transgenic T_2_ lines, along with control lines, were grown in the greenhouse at 22°C under a 16 h light/8 h dark photoperiod.

For MeJA treatment, wheat plants were grown to the tillering stage and fully mature leaves were sprayed with 1 mM MeJA every 3 days until the wheat reached the grain initiation stage. The same amount of sterile water was used as a control (Li et al. 2022).

For the field experiments, the *TaSPL13‐2B* T_9_ transgenic and control plants were grown at two different experimental stations located in Hongshan (114°25′25″ E,30°31′17″ N) and Xinzhou (114°46′30″ E, 30°45′1″ N), Hubei Province, China, from October 2022 to May 2023. Field experiments to measure yield‐related traits were conducted at each location using a completely randomised block design with 20 plots with four replicates. Each plot, which was 1.5 m long and 2 m wide, was sown with 400 seeds in eight rows spaced 0.25 m apart at a density of approximately 133 seeds/m^2^. Two adjacent plots were spaced 0.5 m apart. Spike length, spike weight, grain weight per spike, number of florets per spike, grains per spike, florets per spikelet and grains per spikelet were collected from the 20 main spikes in each plot. The thousand‐grain weight was measured in 20 replicates for each wheat line. The grain yield/m^2^ was determined by the dry grain weight of all plants in the whole plot by the unit area.

### 4.2 Endogenous Phytohormone Measurement

Spikelets were sampled from three positions: the apical (defined as the first to third spikelets from top to bottom), central (the spikelets in the middle of the spike) and basal (the four spikelets counted from the bottom‐most spikelet) positions, of the plants at both the GrA and AN stages for quantification of IAA, GA, CK and JA levels. Upper floret samples from spikelets at the apical, central and basal positions were collected from the wild type plants and two *TaSPL13‐2B* transgenic lines during the floret development stage to determine endogenous JA‐Ile levels. The measurement of endogenous phytohormones was conducted by Metware Biotechnology Co. Ltd. (Wuhan, China).

### 4.3 RNA Extraction and qRT‐PCR Analysis

For gene expression analysis, RNA samples were collected from the roots, stems and leaves at the seedling stage and from the leaf blades, leaf sheaths, nodes, internodes and flag leaves at the booting stage. In addition, data from spikes were collected at seven early stages of development before flowering: the elongation stage (ES), single ridge stage (SR), double ridge stage (DR), differentiation stage (DS), terminal spikelet stage (TS), white anther stage (WA), green anther stage (GrA) and yellow anther stage (YA). Total RNA was isolated with a Total RNA Extraction Kit (Zomanbio, Beijing, China), and cDNA was synthesised using the HiScript III All‐in‐one RT SuperMix Perfect for qPCR Kit (Vazyme, Nanjing, China). The *TaActin* gene served as the internal control.

### 4.4 Plasmid Construction and Transformation

The coding sequence of *TaMADS1* was cloned and inserted into the maize ubiquitin promoter‐driven plasmid pAHC25. The *TaSPL13‐2B* genomic DNA sequence, including its own promoter and terminator sequences, was cloned to generate *TaSPL13‐2B* transgenic lines (Li et al. 2020). The recombinant plasmid was transformed into immature embryos by particle bombardment transformation as previously described (Wang et al. 2022). Transgene‐positive plants were confirmed by herbicide‐resistant selection followed by PCR using specific primers.

### 4.5 In Situ Hybridization

Specific fragments (~200 bp) of *TaSPL13‐2B*, *TaMADS1*, *TaJAZ1* and *TaMYC2* were amplified from wheat cDNA and cloned into the pPST18 and pPST19 vectors using *Hin*dIII and *Eco*RI as probes, respectively. Antisense and sense probes were synthesised and DIG‐labelled using a DIG RNA Labelling Kit (SP6/T7) (Roche, Indianapolis, USA). Samples were collected from early‐stage wheat spikes, including DR, DS and TS. These samples were fixed in 4% (v/v) paraformaldehyde and embedded in paraffin.

### 4.6 Stereomicroscopy and Scanning Electron Microscopy

Spike samples were observed at the early stages of development by stereomicroscopy to temporally characterise the difference in spike morphology between wild type and *TaSPL13‐2B* transgenic line plants. Briefly, the young spikes were carefully detached with tweezers, fixed in 2.5% (v/v) glutaraldehyde overnight at 4°C and photographed under a stereomicroscope. For SEM, young spikes from the wild type plants and *TaSPL13‐2B* transgenic line at the GrA stage were fixed in 2.5% glutaraldehyde overnight at 4°C, dehydrated in a series of ethanol solutions, subjected to critical‐point drying and then replaced with 3‐methylbutyl acetate. The samples were then coated with platinum and observed under a Hitachi S‐3000 N variable pressure scanning electron microscope.

### 4.7 RNA‐Seq and Data Analysis

At the floret development stage, the upper florets of the spikelets from the wild type plants and the transgenic lines were detached and collected. Total RNA was isolated using the RNAprep Pure Plant Kit (Tiangen, Beijing, China), followed by mRNA purification and cDNA library construction. Six libraries were sequenced on the Illumina HiSeq 2000 platform. Raw reads were preprocessed with Cutadapt (version: 1.11) (Martin 2011) to remove adapters and low‐quality bases, and quality was assessed using FastQC (version: 0.11.5) (Andrews 2010). Clean reads were aligned to the wheat reference (IWGSC RefSeq version 1.1) with HISAT2 (version: 2.0.1) (Kim et al. 2015). Gene expression was quantified by FPKM using feature counts. Differentially expressed genes were identified with edgeR (FDR < 0.05, |log2 (Fold Change)| > = 1). GO enrichment analysis was performed using ClusterProfiler (Yu et al. 2012). Sequencing was outsourced to the IGENEBOOK Biotechnology Co. Ltd. (Wuhan, China).

### 4.8 DAP‐Seq and Data Analysis

DAP‐seq analyses were carried out as previously described (Bartlett et al. 2017). Briefly, genomic DNA (gDNA) was extracted from young wheat spikes and used to construct a sequencing library. The recombinant TaSPL13‐2B‐Halo Tag protein was expressed in vitro using a TNT SP6 High Yield Wheat Germ Protein Expression System (Promega, USA). The protein‐bound beads or control beads were incubated with the gDNA library, followed by washing and elution of the bound DNA. Libraries were quantified and sequenced on an Illumina Nova S4 platform using 150 bp paired‐end reads (Illumina, USA).

For data analysis, the raw reads were quality‐filtered with Trimmomatic (version: 0.36) and assessed by FastQC (version: 0.11.5) (Andrews 2010). Clean reads were aligned to the wheat genome (IWGSC RefSeq v1.1) using BWA (version: 0.7.15‐r1140) (Li and Durbin 2009). Genome‐wide analysis of the DAP‐seq peak information was performed using MACS2 (version: 2.1.2) (Zhang et al. 2008) with a *q* value threshold < 0.05 to filter out significant peaks. HOMER (version: 4.11) (Heinz et al. 2010) was used for motif enrichment analysis with default parameters.

To process publicly available DAP‐seq data, the dataset was downloaded from NCBI and annotated by using the R package ChIPseeker. Gene IDs were converted using the ID converter function from the WheatOmics web portal. GO enrichment analysis was performed as described for the RNA‐seq analysis.

### 4.9 Yeast One‐Hybrid Assay

Four fragments containing a potential TaSPL13‐2B binding motif were identified in the *TaJAZ1* promoter, and three fragments containing a G‐box motif were selected from the *TaMADS1* promoter. These fragments were amplified and inserted into pAbAi (Clontech, Japan) via *Hin*dIII and *Sac*I digestion to construct bait The linearized pAbAi bait was digested with *Bst*BI and transformed into the *S. cerevisiae* strain *Y1HGold* (Clontech, Japan) and spread on SD/‐Ura medium. Positive strains were confirmed by PCR and screened with different concentrations (50–1000 ng/mL) of Aureobasidin A (AbA, Coolaber, Beijing, China) on SD/‐Ura/AbA plates to determine the appropriate concentration for the elimination of self‐activation.

The coding sequences of *TaSPL13‐2B* and *TaMYC2* were ligated into the pGADT7 vector (Clontech, Japan) after digestion at the *Eco*RI and *Bam*HI sites to use as prey. The recombined plasmids were transformed into positive bait strains and spread on SD/‐Ura/−Leu media supplemented with an appropriate concentration of AbA. Protein–DNA interactions were determined on the basis of the growth ability of transformed yeast cells on SD/‐Ura/−Leu/AbA medium after 3 days at 28°C.

### 4.10 Yeast Two Hybrid and Transactivation Assays

The recombinant constructs were cotransformed into the *AH109* strain (Clontech, Japan) and then cultivated at 30°C for 4 days on SD/−Trp/−Leu medium. Detection of protein–protein interactions between TaJAZ1 and TaMYC2 was performed on SD/−Trp/−Leu/−His/−Ade media. The full‐length or truncated domain of *TaMYC2* was subsequently inserted into pGBKT7 with *Bam*HI and *Eco*RI digestion and ligation. The recombinant plasmids were subsequently transformed into yeast strain *AH109*, which was cultured on SD/−Trp, SD/−Trp/−Ade, SD/−Trp/−His and SD/−Trp/−His/−Ade media at 30°C for 3–4 days.

### 4.11 Bimolecular Fluorescence Complementation and Subcellular Localization Assays

To generate the BiFC vectors, the full‐length sequences of *TaMYC2* and *TaJAZ1* were subcloned into pSPYNE (N‐terminal of YFP) and pSPYCE (C‐terminal of YFP) vectors with *Sma*I and *Bam*HI restriction sites, respectively. The coding sequences of *TaJAZ1*, *TaMYC2* and *TaMADS1* were constructed in pBI121‐eGFP under the CaMV 35S promoter using *Xba*I and *Bam*HI restriction sites. The recombinant constructs and empty vectors were individually introduced into *Agrobacterium* strain *GV3101* (*pSoup‐p19*) (WeiDi, Shanghai, China). After being cultured at 28°C for 18 h, the *Agrobacteria* carrying the constructs were collected by centrifugation and resuspended in infiltration buffer (10 mM MES, 10 mM MgCl_2_ and 150 μM acetosyringone, pH 5.6) to a final OD_600_ of 1. After 3 h, the *Agrobacteria* were injected into young tobacco leaves. After 36–48 h of incubation, the fluorescence signals were visualised using a fluorescence microscope (Olympus Lx71, Japan). Coexpression of pSPYCE‐TaJAZ1 and pSPYNE was used as a negative control. At least three leaves were analysed, with similar results.

### 4.12 Co‐Immunoprecipitation Assay

Procedures and vectors used for transient transformation in *N*. *benthamiana* leaves were described in the BiFC analysis protocol. After 48–60 h of incubation, NP‐40 lysis buffer containing 1 mM PMSF (Beyotime, Shanghai, China) was used to extract total proteins. The extracted proteins were incubated with 1 μg monoclonal anti‐HA antibody (MBL, Japan) for 16 h on a rotary shaker at 4°C. Co‐IP was performed using a Capturem IP & Co‐IP Kit (Takara, Dalian, China). The samples were analysed by western blotting using an anti‐Myc antibody (MBL, Japan).

### 4.13 GST Pull Down Assay

The coding sequence of *TaMYC2* was cloned into the pET‐28a (+) vector with *Hin*dIII and *Nde*I, carrying the maltose binding protein (MBP) CDS in‐frame and upstream of *TaMYC2*, resulting in His‐MBP‐*TaMYC2*. For the GST‐tagged proteins, the full‐length sequence of *TaJAZ1* or 6 × His was inserted into the pGEX‐4 T‐1 vector using the *Bam*HI and *Eco*RI restriction sites, resulting in the construction of GST–TaJAZ1 and GST–His.

The recombinant constructs and empty vector were introduced into *Escherichia coli Rosetta* (*DE3*) competent cells (WeiDi, Shanghai, China). The recombinant proteins were induced with 1 mM isopropyl‐β‐D‐thiogalactoside (IPTG) at 16°C for 20–24 h. The culture was pelleted and resuspended in 50 mL of ice‐cold binding buffer (140 mM NaCl, 2.7 mM KCl, 10 mM Na_2_HPO_4_, 1.8 mM KH_2_PO_4_, 5 mM DTT and pH 7.4), and the supernatant was collected after centrifugation. GST‐tagged proteins (GST‐TaJAZ1 and GST‐His) and MBP‐fused protein (MBP–TaMYC2–His) were purified using Glutathione Agarose Resin and MBP‐Sep Dextrin Agarose Resin 6FF (Yeasen, Shanghai, China) according to the manufacturer's instructions.

For the pull‐down assay, 10 μg of GST–His or GST–TaJAZ1 was bound to glutathione agarose resin and directly incubated overnight with 10 μg His–MBP–TaMYC2 in PBS at 4°C, after which the beads were washed seven times with wash buffer. After the beads were washed, the protein complex was eluted with 50 μL of elution buffer and then analysed by immunoblotting with anti‐GST antibody (CWBIO, Beijing, China) or anti‐His antibody (MBL, Japan).

### 4.14 Detection of Protein Degradation In Vivo

The full‐length *TaJAZ1* coding sequence was cloned into the pGreenII 0080‐LUC vector under the control of the CaM 35S promoter for luciferase assays. For western blot analysis, *TaJAZ1* fused with an HA tag was cloned into the pBI121 vector. *Agrobacterium*‐mediated transient expression in *N*. *benthamiana* leaves was performed as described for the BiFC assays. After 48 h, the leaves were treated with 100 μM MeJA (Macklin, Shanghai, China) for 1 h or cotreated with both MeJA and the proteasome inhibitor MG‐132 (Beyotime, Shanghai, China). For luciferase imaging, the leaves were injected with 1 mM of D‐luciferin sodium salt (Yeasen, Shanghai, China) and then imaged using a GelView6000proII Chemiluminescent imaging system with a 50 s exposure time. To perform immunoblotting, total proteins were extracted and probed with an anti‐HA antibody (MBL, Japan).

### 4.15 Electrophoretic Mobility Shift Assay

The coding sequence of *TaSPL13‐2B* fused to MBP was cloned into the pCold‐MBP vector. MBP–TaSPL13‐2B and pCold‐MBP were purified as previously described in the GST pull‐down assay, which was consistent with that of the MBP‐fused proteins. Oligonucleotide probes were synthesised and labelled with biotin by AuGCT Biological Technology (Beijing, China). Double‐stranded DNA probes were generated by annealing the complementary oligonucleotides. The EMSA was performed using a LightShift Chemiluminescent EMSA Kit (Thermo Fisher Scientific, Waltham, USA) according to the manufacturer's instructions, with some modifications.

### 4.16 Dual Luciferase Assay

For effector construction, the coding regions of *TaSPL13‐2B, TaMYC2* and *OsSPL13* were cloned into the pGreenII 62 SK vector under the CaMV35S promoter. To construct the reporters, the promoter sequences of *TaJAZ1*, *TaMADS1*, *TaOPR12*, *TaJAR1* and *OsJAZ1*, as well as their deleted versions lacking the TaSPL13‐2B binding motif, were inserted into pGreenII 0080‐LUC.

Transient expression was performed in tobacco leaves via *Agrobacterium*‐mediated transformation (refer to the BiFC assays section). For JA treatment, 50 mM MeJA was injected at 36 h posttransformation. Firefly luciferase (LUC) and Renilla luciferase (REN) activities were quantified using a dual‐luciferase reporter kit (Yeasen, Shanghai, China) on a FlexStation3 system (250–850 nm). To normalise the data, the luminescence readings of LUC and REN from both the experimental and control groups were adjusted by subtracting the values obtained from the black control group. Afterward, the normalised LUC readings were divided by the REN readings to obtain the ratios for both the experimental and control groups. The fold change in expression was calculated by dividing the ratio of the experimental group by that of the control group. The pGreenII 62 SK vector served as the negative control.

### 4.17 Statistical Analysis

Statistical analyses were performed using Microsoft Excel and visualised using GraphPad Prism v8 for bar graphs, and OriginPro 2019b for violin plots. Details of the statistical parameters, including the means ± SDs (standard deviations) and the number (*n*) of samples or biological replicates, are described in the corresponding figure legends. Two‐tailed Student's *t*‐test was used for statistical comparison. Exact *p* values are provided, and asterisks indicate levels of statistical significance unless otherwise stated: * indicates *p* < 0.05; ** indicates *p* < 0.01; *** indicates *p* < 0.001.

All primers used for vector construction, probe sequences, gene expression and gene amplification are provided in Table S10.

## Author Contributions

Yin Li, Guangyuan He and Yuesheng Wang conceived and designed the research. Li Li and Fu Shi performed most of the experiments, analysed the data and drafted the manuscript. Li Li, Fu Shi and Yaqiong Wang performed the genetic transformation. Li Li, Fu Shi and Yanbin Guan performed the phenotypic analysis and statistics. Fu Shi and Ya'nan Wu participated in the analysis of DAP‐seq and RNA‐seq data. Ling Chen helped with the field experiment. Junli Chang, Mingjie Chen and Ling Chen performed project administration and provided valuable comments. Li Li and Fu Shi wrote the paper. Yin Li, Guangyuan He, Yuesheng Wang, Guangxiao Yang and Jun Xiao revised the paper. All authors have read and approved the published version of the manuscript. Li Li and Fu Shi contributed equally to this work.

## Disclosure

Accession Numbers: Sequence data were obtained from the EnsemblPlants database (http://plants.ensembl.org/Triticum_aestivum/Info/Index) using the following accession numbers: *TaSPL13‐2B* (TraesCS2B02G250900), *TaJAZ1* (TraesCS2D02G507200), *TaCoI3‐4A* (TraesCS4A02G091200), *TaMYC2* (TraesCS1A02G193200), *TaMADS1* (TraesCS4A02G028200), *TaOPR12* (TraesCS2B02G328200) and *TaJAR1* (TraesCS1A02G425100).

## Conflicts of Interest

The authors declare no conflicts of interest.

## Supporting information


**Figure S1:** Measurement of endogenous phytohormone and transcriptome data analysis of JA‐responsive genes and spike developmental stages.
**Figure S2:** ATAC‐seq analysis and sequence alignment of the *TaSPL13* gene promoters.
**Figure S3:** Alignment of *TaSPL13* cDNA from the A, B and D subgenomes.
**Figure S4:** Comparison of TaSPL13 amino acid sequences from the A, B and D subgenomes.
**Figure S5:** qRT‐PCR analysis of *TaSPL13‐2B* expression levels.
**Figure S6:** *TaSPL13‐2B* transgenic lines increase floret primordia fertility.
**Figure S7:** TaSPL13‐2B increases the expression of JA response marker genes.
**Figure S8:** Genome‐wide identification of TaSPL13‐2B binding sites by DAP‐seq.
**Figure S9:** Transcriptome sequencing of *TaSPL13‐2B* transgenic lines and wild type.
**Figure S10:** TaJAZ1 interacts with TaCOI3‐4A and is degraded via the 26S proteasome.
**Figure S11:** Expression patterns of *TaJAZ1* and *TaMYC2* genes and their protein interactions.
**Figure S12:** Subcellular localization of TaMYC2, TaJAZ1 and TaMADS1.
**Figure S13:** *TaMADS1* is upregulated in the spikelets of *TaSPL13‐2B* transgenic wheat.
**Figure S14:** Transcriptional activity analysis of the TaMYC2 protein.
**Figure S15:** OsSPL13 inhibits *OsJAZ1* expression.
**Figure S16:** Field performance of *TaSPL3‐2B* T_8_ transgenic lines in Hongshan, Hubei Province, China from October 2021 to May 2022.
**Figure S17:** Statistical comparison of *TaSPL13‐2B* transgenic and control lines in the 2022/23 field season at the Hongshan experimental field.
**Figure S18:** Field experiments show improved yield traits in three *TaSPL13‐2B* transgenic lines versus controls during the 2022/23 field season at the Xinzhou experiment field, with a randomised block design.
**Figure S19:** Distribution of florets and grains per spikelet at apical, central and basal positions on a single wheat spike.

**Table S1:** Integration of information from 488 genes screened by RNA‐seq from JA‐treated spikes and different stages of spikes development.
**Table S2:** GO enrichment analysis of the 488 genes.
**Table S3:** Phenotypic analysis of T_7_ transgenic *TaSPL13‐2B* plants in the greenhouse.
**Table S4:** Motif enrichment analysis from DAP‐seq data.
**Table S5:** Go enrichment analysis combining DAP‐seq and down‐regulated gene expression.
**Table S6:** Expression levels of *TaJAZ* genes in the wheat spikes.
**Table S7:** Phenotypic analysis of T_8_ transgenic *TaSPL13‐2B* plants in the field in Hongshan, Hubei Province, China from October 2021 to May 2022.
**Table S8:** Phenotypic analysis of T_9_ transgenic *TaSPL13‐2B* plants in the field in Hongshan, Hubei Province, China from October 2022 to May 2023.
**Table S9:** Phenotypic analysis of T_9_ transgenic *TaSPL13‐2B* plants in the field in Xinzhou, Hubei Province, China from October 2022 to May 2023.
**Table S10:** Primers used in this study.

## Data Availability

The data that support the findings of this study are available in Figures S1–S19 and Tables S1–S10. The RNA‐seq and DAP‐seq data have been deposited to the NCBI with accession numbers PRJNA1130840 and PRJNA1130865, respectively.
