# Supplementary material for: Wheat TaSPL13‐2B Improves Floret Fertility and Enhances Grain Number per Spikelet Through Jasmonic Acid Signalling Pathway
Source: Plant Biotechnol J. 2025 Nov 19;24(4):2056–75. doi: 10.1111/pbi.70463 (PMC13140700; doi:10.1111/pbi.70463)
Supplement: Supplementary file 1 — Figure S1: Measurement of endogenous phytohormone and transcriptome data analysis of JA‐responsive genes and spike developmental stages. Figure S2: ATAC‐seq analysis and sequence alignment of the TaSPL13 gene promoters. Figure S3: Alignment of TaSPL13 cDNA from the A, B and D subgenomes. Figure S4: Comparison of TaSPL13 amino acid sequences from the A, B and D subgenomes. Figure S5: qRT‐PCR analysis of TaSPL13‐2B expression levels. Figure S6: TaSPL13‐2B transgenic lines increase floret primordia fertility. Figure S7: TaSPL13‐2B increases the expression of JA response marker genes. Figure S8: Genome‐wide identification of TaSPL13‐2B binding sites by DAP‐seq. Figure S9: Transcriptome sequencing of TaSPL13‐2B transgenic lines and wild type. Figure S10: TaJAZ1 interacts with TaCOI3‐4A and is degraded via the 26S proteasome. Figure S11: Expression patterns of TaJAZ1 and TaMYC2 genes and their protein interactions. Figure S12: Subcellular localization of TaMYC2, TaJAZ1 and TaMADS1. Figure S13: TaMADS1 is upregulated in the spikelets of TaSPL13‐2B transgenic wheat. Figure S14: Transcriptional activity analysis of the TaMYC2 protein. Figure S15: OsSPL13 inhibits OsJAZ1 expression. Figure S16: Field performance of TaSPL3‐2B T8 transgenic lines in Hongshan, Hubei Province, China from October 2021 to May 2022. Figure S17: Statistical comparison of TaSPL13‐2B transgenic and control lines in the 2022/23 field season at the Hongshan experimental field. Figure S18: Field experiments show improved yield traits in three TaSPL13‐2B transgenic lines versus controls during the 2022/23 field season at the Xinzhou experiment field, with a randomised block design. Figure S19: Distribution of florets and grains per spikelet at apical, central and basal positions on a single wheat spike. [file PBI-24-2056-s001.docx]

**Supporting information**

**Wheat TaSPL13-2B improves floret fertility and increases grain number per spikelet through jasmonic acid signaling pathway**

Li Li^1†^, Fu Shi^1†^, Yaqiong Wang^1^, Yanbin Guan^1^, Ya’nan Wu^1^, Ling Chen^5,6,7^, Junli Chang^1^, Mingjie Chen^1^, Jun Xiao^2,3,4^, Guangxiao Yang^1^, Yuesheng Wang^1^*, Guangyuan He^1^*, and Yin Li^1^*

^1^ The Genetic Engineering International Cooperation Base of Chinese Ministry of Science and Technology, the Key Laboratory of Molecular Biophysics of Chinese Ministry of Education, College of Life Science and Technology, Huazhong University of Science & Technology, Wuhan 430074, China.

^2^ Key Laboratory of Plant Cell and Chromosome Engineering, Institute of Genetics and Developmental Biology, Chinese Academy of Sciences, Beijing 100101, China.

^3^ University of Chinese Academy of Sciences, Beijing 100049, China.

^4^ CAS-JIC Centre of Excellence for Plant and Microbial Science (CEPAMS), Institute of Genetics and Developmental Biology, Chinese Academy of Sciences, Beijing 100101, China.

^5^ Institute of Food Crops, Hubei Academy of Agricultural Sciences, Wuhan 430064, China.

^6^ Hubei Key Laboratory of Food Crop Germplasm and Genetic Improvement, Wuhan 430064, China.

^7^ Key Laboratory of Crop Molecular Breeding, Ministry of Agriculture and Rural Affairs, Wuhan 430064, China.

* Corresponding authors: Yuesheng Wang (wysh@hust.edu.cn), Guangyuan He (hegy@hust.edu.cn); Yin Li (yinli2021@hust.edu.cn).


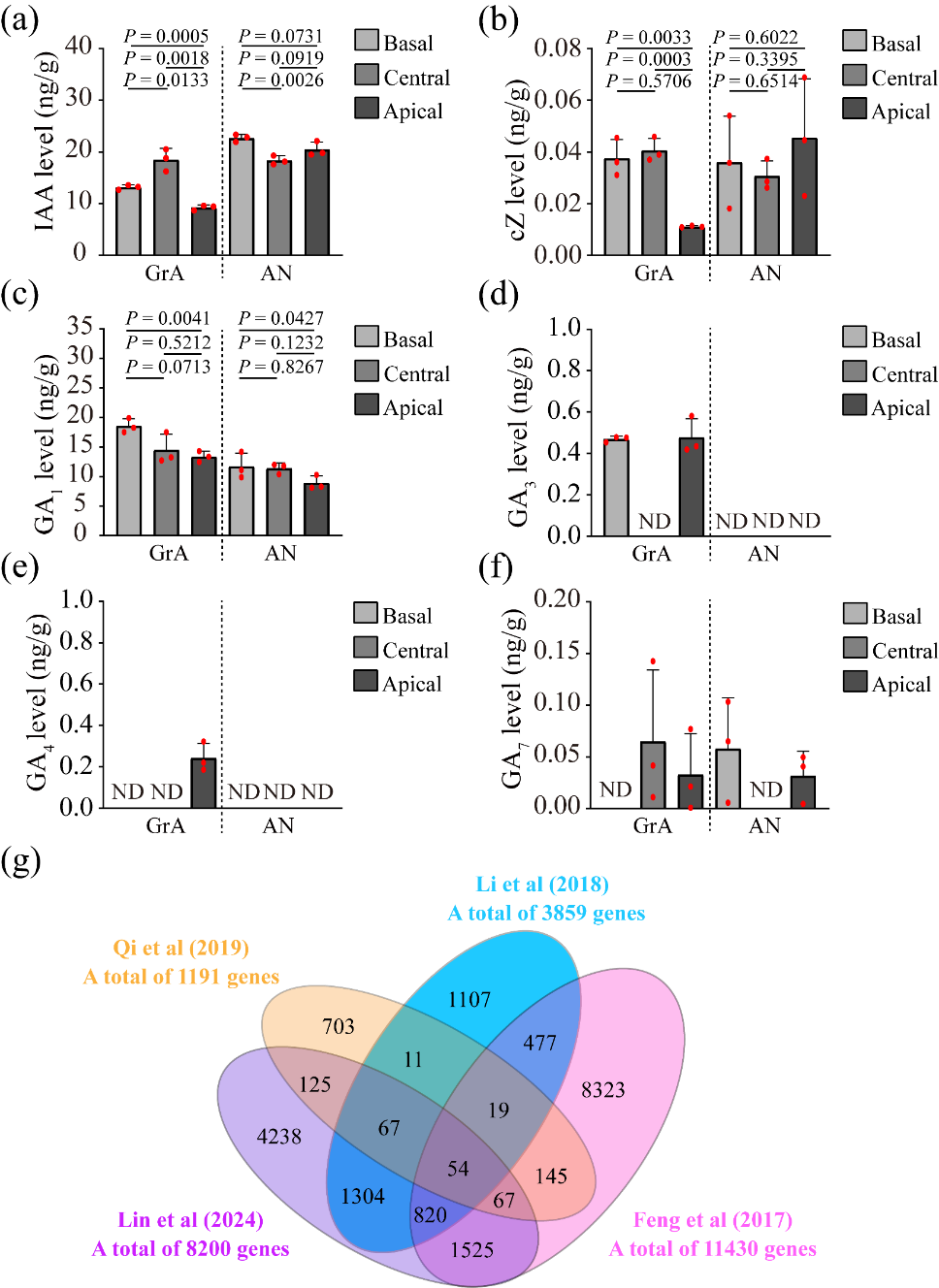


**Figure S1** Measurement of endogenous phytohormone and transcriptome analysis of JA-responsive genes and spike developmental stages. (a-f) Measurement of endogenous phytohormone metabolite levels of IAA (a), cis-Zeatin (cZ) (b), GA_1_ (c), GA_3_ (d), GA_4_ (e), and GA_7_ (f) in spikelets at apical, central, and basal positions. GrA: green anther stage; AN: anthesis stage; ND: not detected. n=3 biological replicates. Dots represent data distribution. Exact *P* values were determined using a two-tailed Student’s *t*-test are shown in the figures. (g) Venn diagram showing the overlap of genes among datasets from Qi’s (yellow oval) (Qi *et al.,* 2019), Li’s (green oval) (Li *et al.,* 2018), Lin’s (violet oval) (Lin *et al.,* 2024), and Feng’s (red oval) (Feng *et al.,* 2017).


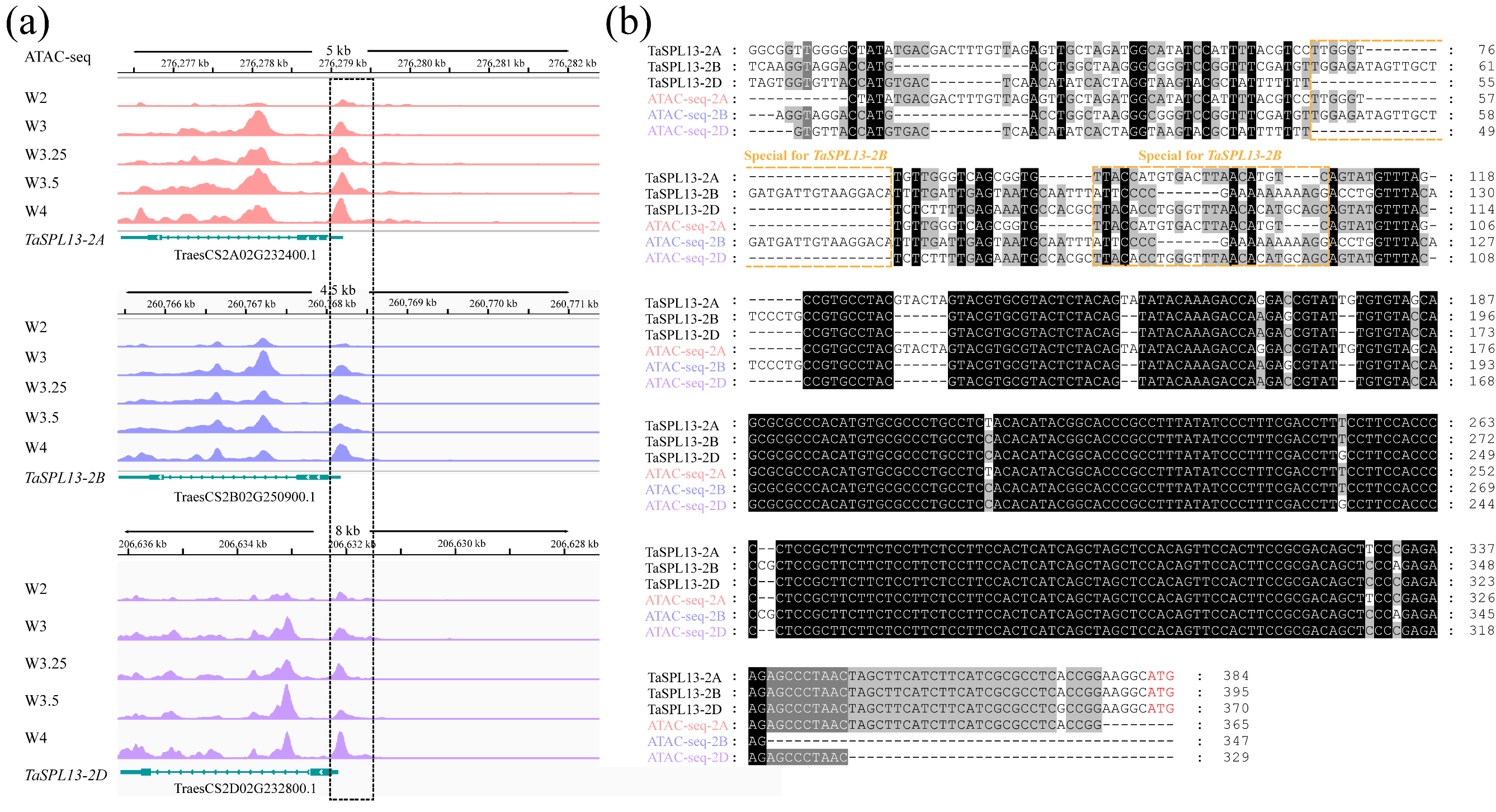


**Figure S2** ATAC-seq analysis and sequence alignment of the *TaSPL13* promoters. (a) ATAC-seq analysis of *TaSPL13* genomic sequences at different young spike development stages. Blank dashed rectangle indicate the promoter regions of *TaSPL13* genes (Lin *et al.,* 2024); the arrow shows the direction of the gene. (b) Multiple alignment of the three homoeologous *TaSPL13* promoters. Yellow dashed rectangles indicate sequences unique to *TaSPL13-2B*. Base highlighted in red correspond to the start codon ATG.


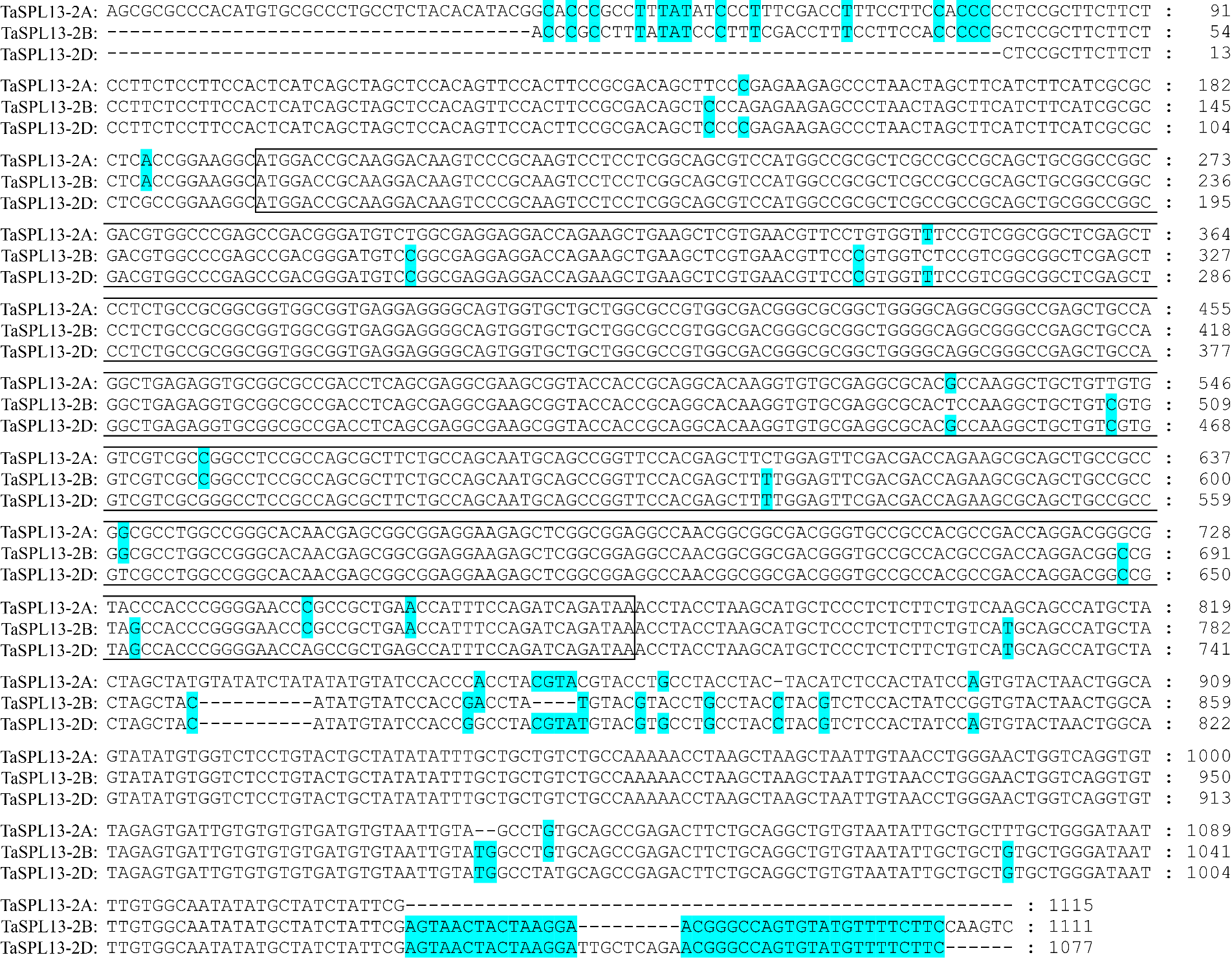
**Figure S3** Alignment of *TaSPL13* cDNA from the A, B, and D subgenomes. The black rectangle denotes the coding sequence (CDS); flanking regions represent the 5′ and 3′ UTRs.


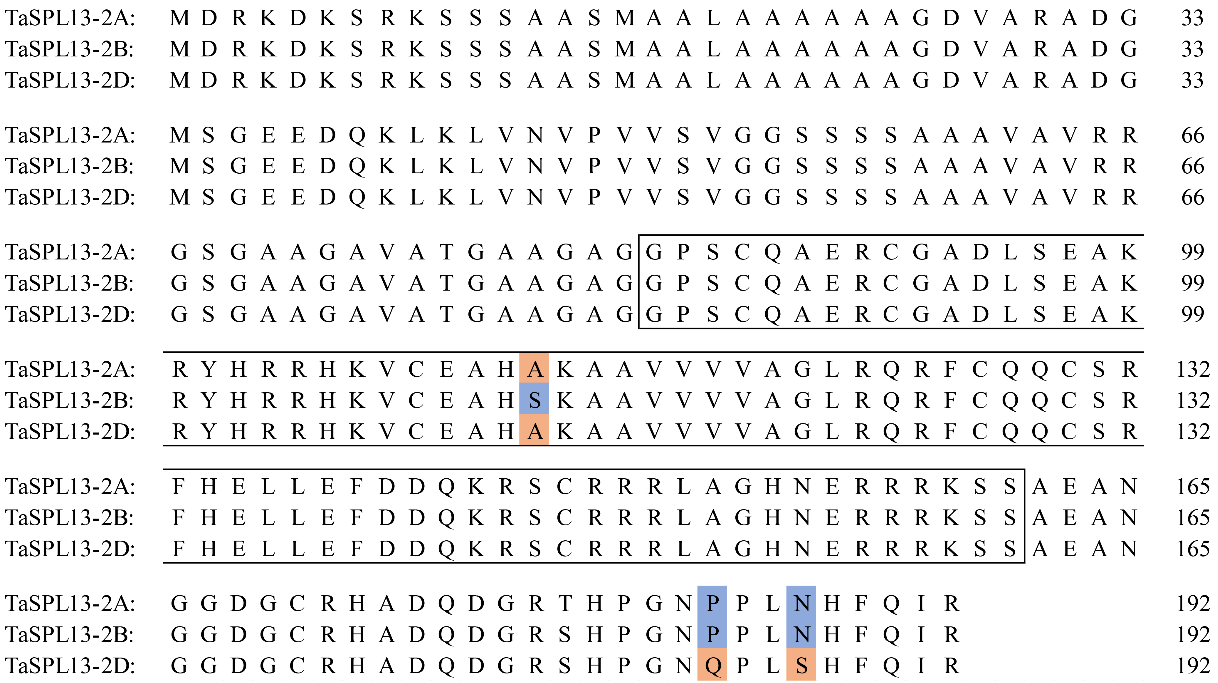


**Figure S4** Comparison of TaSPL13 amino acid sequences from the A, B, and D subgenomes. The black rectangle marks the conserved SBP domain.


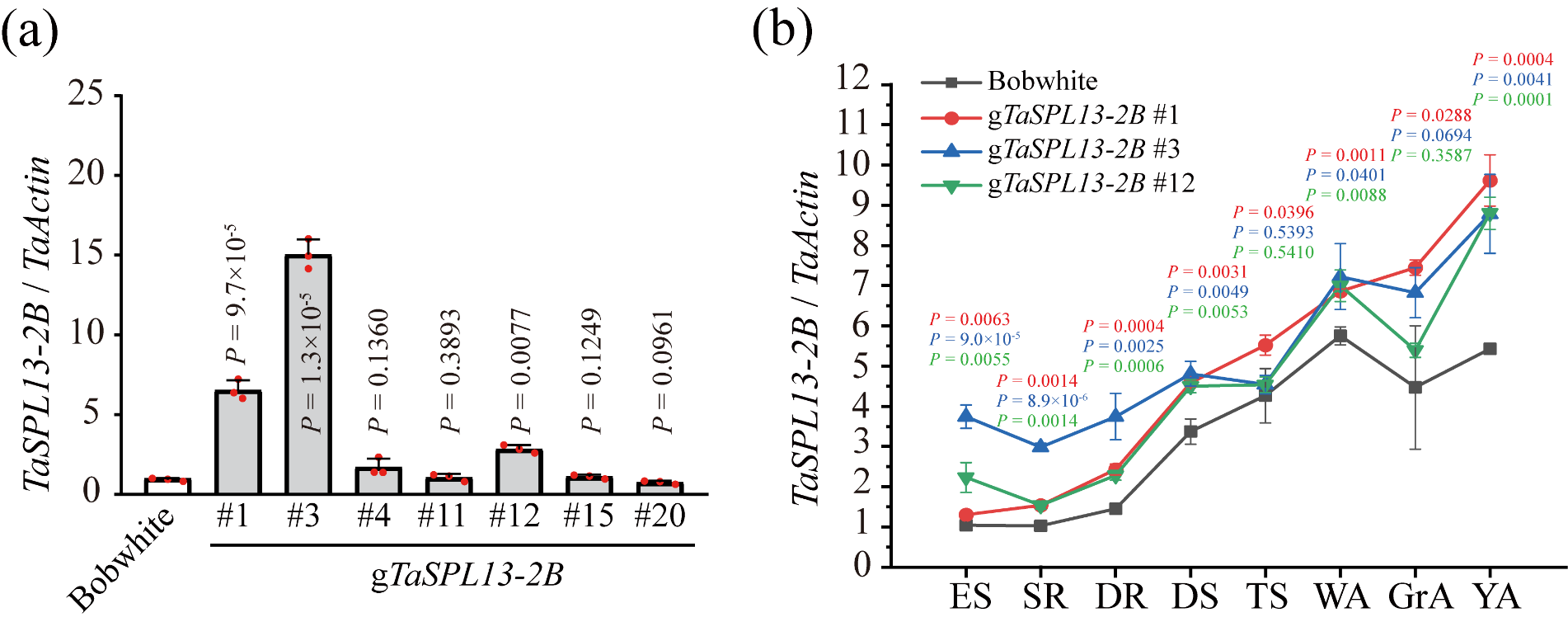


**Figure S5** qRT-PCR analysis of *TaSPL13-2B* expression levels. (a) Relative transcript levels of *TaSPL13-2B* in transgenic lines (#1, #3, #4, #11, #12, #15, and #20). (b) *TaSPL13-2B* expression in transgenic lines and wild type plants during spike development. ES: elongation stage; SR: single ridge stage; DR: double ridge stage; DS: differentiation stage; TS: terminal spikelet stage; WA: white anther stage; GrA: green anther stage; YA: yellow anther stage. Data are means ± SD. Two-tailed Student’s *t*-test was used to determine significant differences. The exact *P* value for each comparison is provided in the corresponding figure.


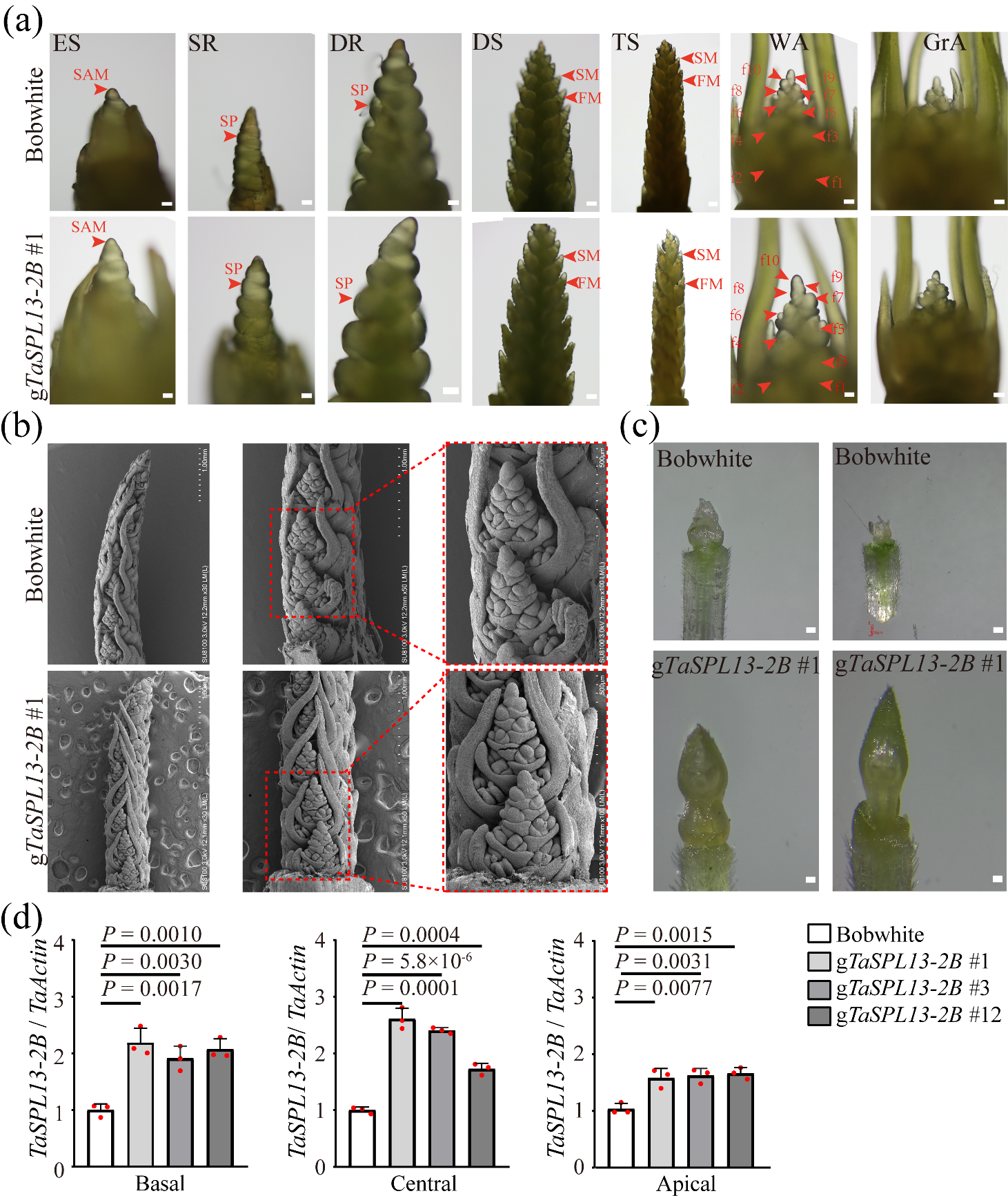


**Figure S6** *TaSPL13-2B* transgenic lines increase floret primordia fertility. (a) Morphology comparison between wild type and g*TaSPL13-2B* #1 at seven early spike development stages. ES: elongation stage; SR: single ridge stage; DR: double ridge stage; DS: differentiation stage; TS: terminal spikelet stage; WA: white anther stage; GrA: green anther stage. SAM: shoot apical meristem; SP: spikelet primordium; SM: spikelet meristem; FM: floret meristem; f: florets. Scale bars: 50 μm. (b) Scanning electron micrographs of young spikes at the GrA between wild type and g*TaSPL13-2B* #1 plants. Scale bars: 100 μm and 500 μm. (c) Images show significant morphology differences in apical florets per spikelet between g*TaSPL13-2B* #1 and wild type plants. Scale bars: 100 μm. (d) qRT-PCR analysis of *TaSPL13-2B* expression in upper florets of spikelets at basal, central, and apical positions in wild type and three transgenic lines. Data are means ± SD. Significant differences were determined using a two-tailed Student’s *t*-test. Dots show the data distribution, exact *P* value for each comparison is provided in each figure.


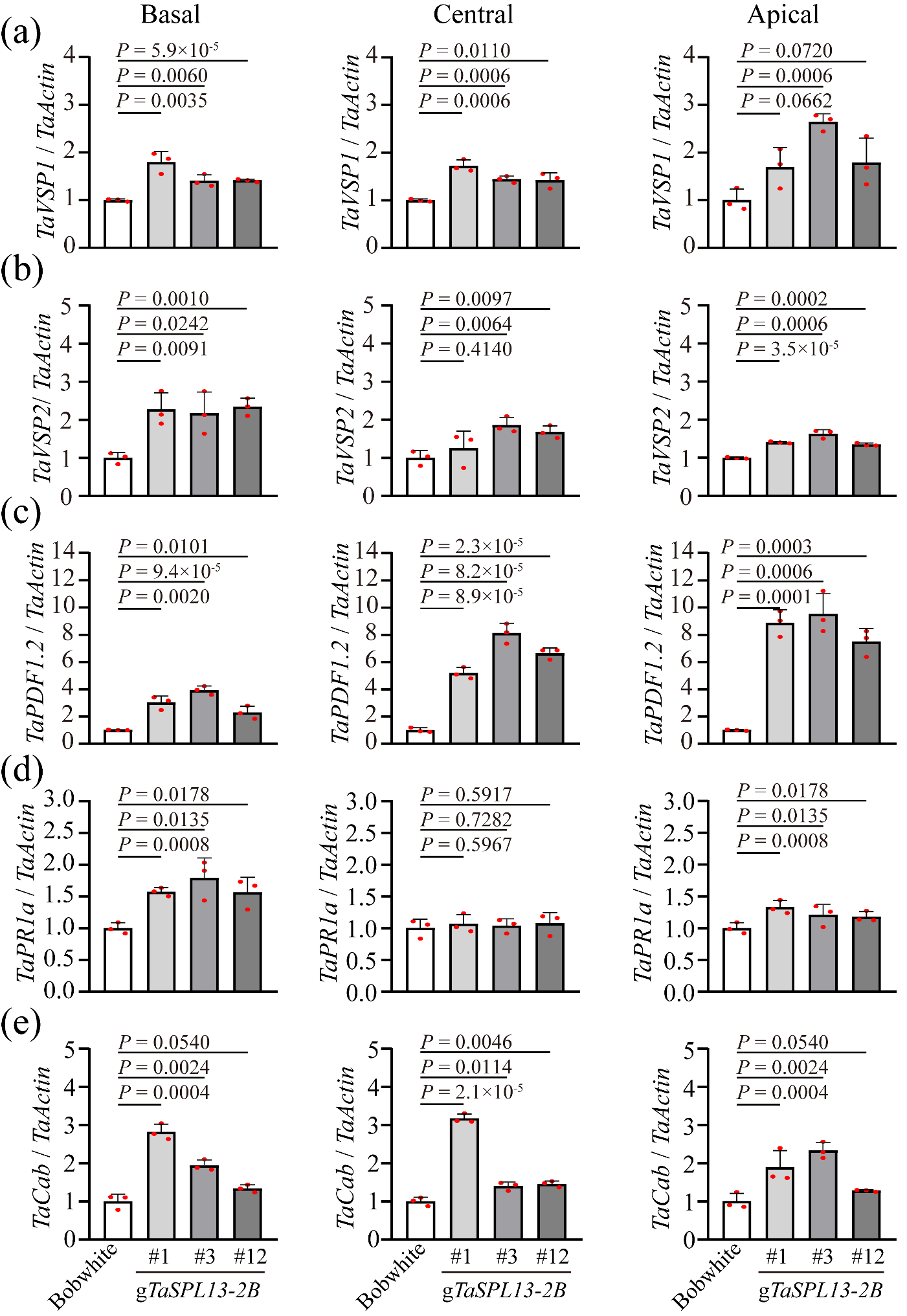


**Figure S****7** TaSPL13-2B increases the expression of JA response marker genes. qRT-PCR analysis of *TaVSP1* (TraesCS4A02G431300) (a), *TaVSP2* (TraesCS7D02G365400) (b), *TaPDF1.2* (TraesCS5A02G330100) (c), *TaPR1a* (TraesCS5D02G446800) (d), and *TaCab* (*chlorophyll a-b binding protein-encoding*, TraesCS6D02G152700) (e) genes expression in the upper floret of spikelets at basal, central, and apical positions in wild type and *TaSPL13-2B* transgenic lines. A two-tailed Student’s *t*-test was used to determine significant differences. Dots show the data distribution, and exact *P* values for each comparison are provided in the figures.


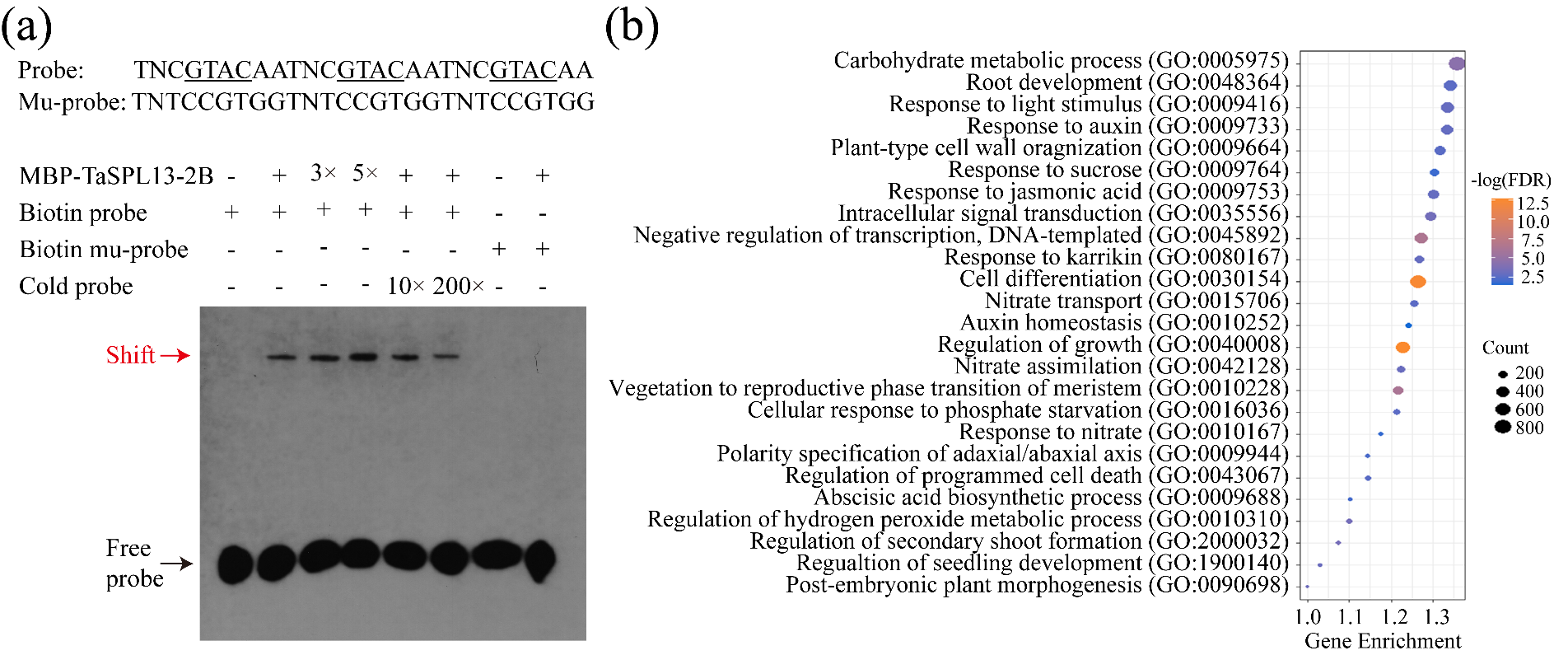


**Figure S8** Genome-wide identification of TaSPL13-2B binding sites by DAP-seq. (a) EMSA showing that TaSPL13-2B directly bound to GTAC-containing probes. Arrows indicate free probes and protein‒DNA complexes; “+” and “-” indicate presence or absence of the corresponding component, respectively. (b) GO enrichment analysis of TaSPL13-2B target genes using our and Lu’s DAP-seq data (Pei *et al.,* 2023). Terms with FDR < 0.05 were considered significantly enriched.


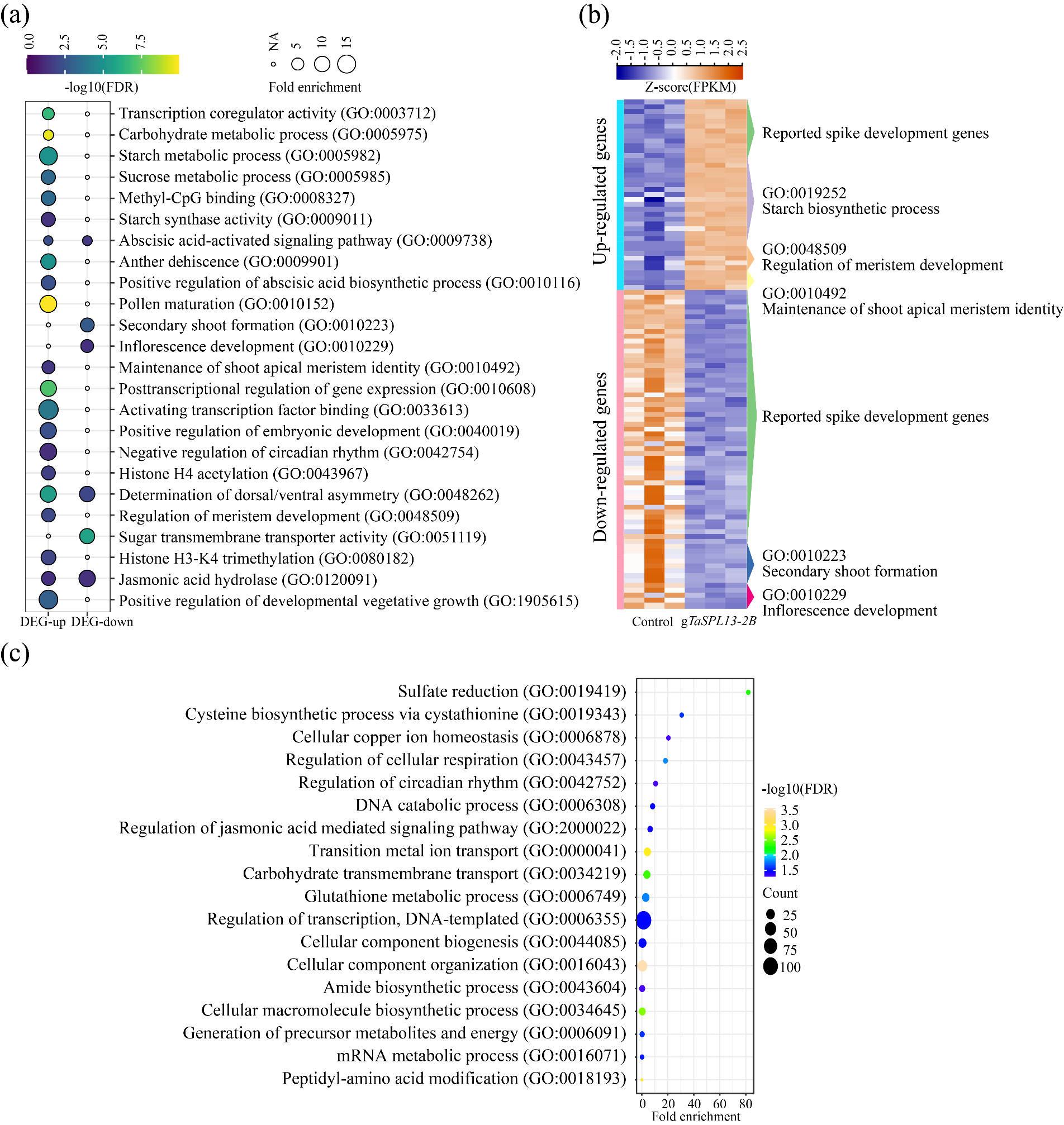


**Figure S9** Transcriptome sequencing of *TaSPL13-2B* transgenic lines and wild type. (a) GO enrichment analysis of differentially expressed genes using bubble map. (b) GO enrichment analysis of differentially expressed genes using heat map. (c) GO enrichment analysis of down-regulated differential genes using bubble map. Relative expression levels of genes in DEG data were calculated using –log 10 (FDR) in (a) and (c), and Z-score (FPKM) in (b).


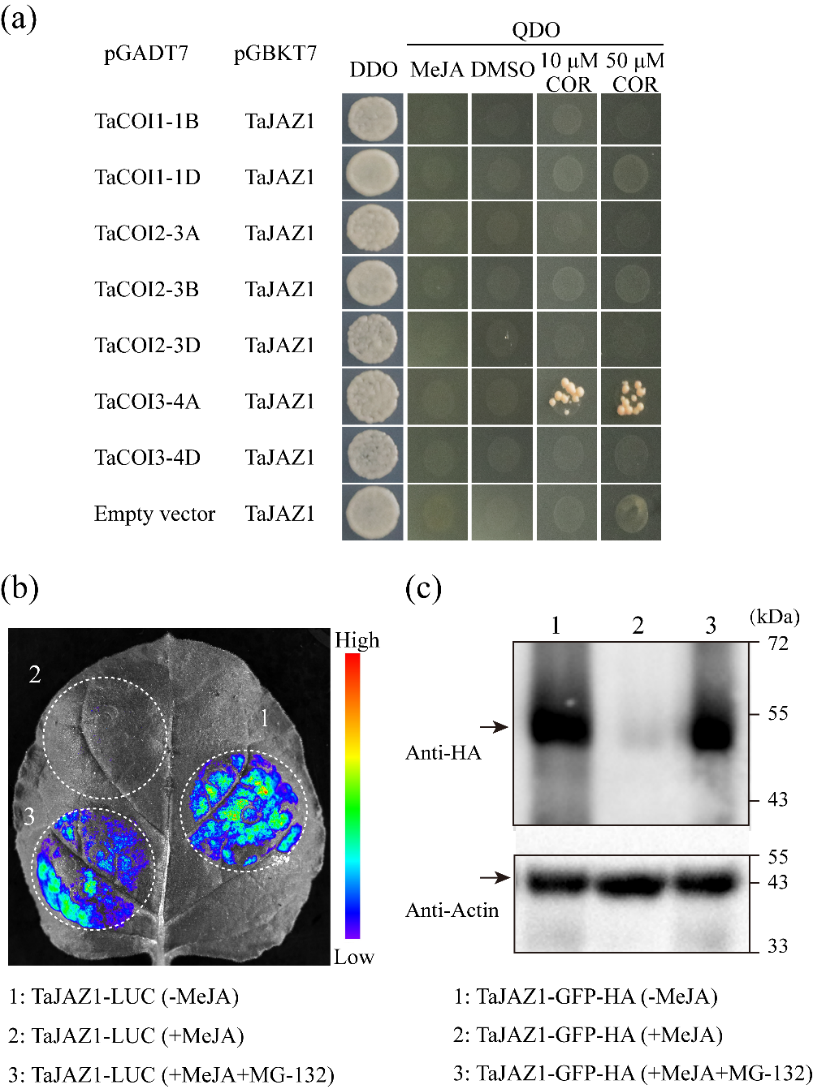


**Figure S10** TaJAZ1 interacts with TaCOI3-4A and is degraded via the 26S proteasome. (a) Y2H assays to test interaction between TaJAZ1 and seven TaCOI proteins. TaJAZ1 interacted with TaCOI3-4A (TraesCS4A02G091200) only when COR was supplied; no interaction was observed with MeJA and DMSO. No Y2H signals were detected between TaJAZ1 and TaCOI1-1B (TraesCS1B02G288100), TaCOI1-1D (TraesCS1D02G278400), TaCOI2-3A (TraesCS3A02G367500), TaCOI2-3B (TraesCS3B02G399200), TaCOI2-3D (TraesCS3D02G360400), or TaCOI3-4D (TraesCS4D02G214200) under any condition. (b) JA-dependent proteasome mediated degradation of TaJAZ1-LUC in *N. benthamiana*. MG-132, a proteasome inhibitor. (c) Immunoblotting assays were performed to assess TaJAZ1 protein accumulation under MeJA or MG-132 treatment. Arrows indicate TaJAZ1-GFP-HA and Actin protein bands, respectively.


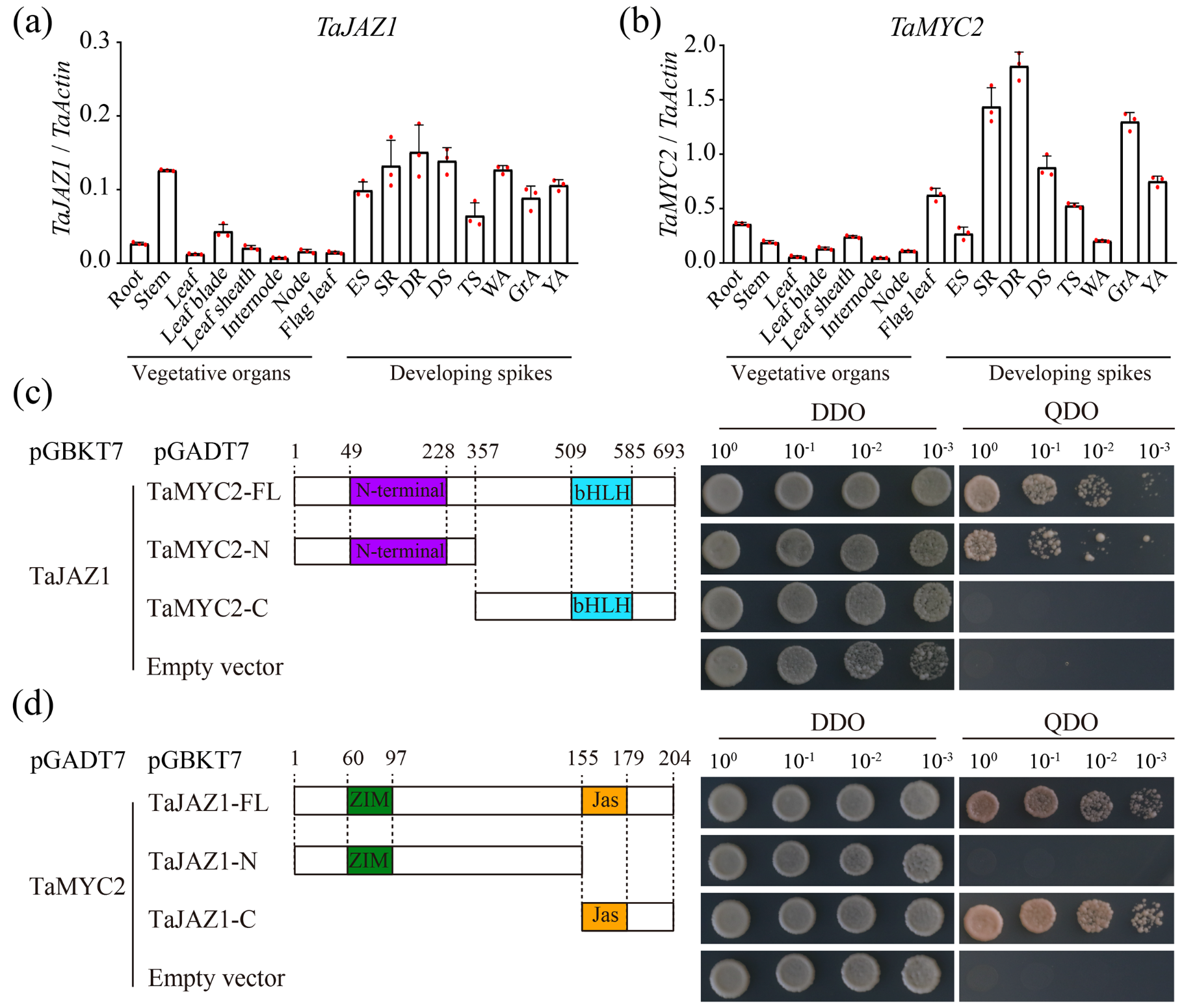


**Figure S11** Expression patterns of *TaJAZ1* and *TaMYC2* genes and their protein interactions. (a-b) Transcriptional profiles of *TaJAZ1* (a) and *TaMYC2* (b). Dots indicate data distribution. ES: elongation stage; SR: single ridge stage; DR: double ridge stage; DS: differentiation stage; TS: terminal spikelet stage; WA: white anther stage; GrA: green anther stage; YA: yellow anther stage. (c) Full-length and truncated TaMYC2 fragments cloned into the pGADT7 vectors are shown on the left. Purple rectangle: N-terminal region; Blue rectangle: bHLH domain. (d) Full-length and truncated *TaJAZ1* fragments cloned into pGBKT7 vector are shown on the left. Green rectangle: ZIM structure; Orange rectangles: Jas domain. DDO: SD/-Trp/-Leu medium; QDO: SD/-Trp/-Leu/-Ade/-His medium.


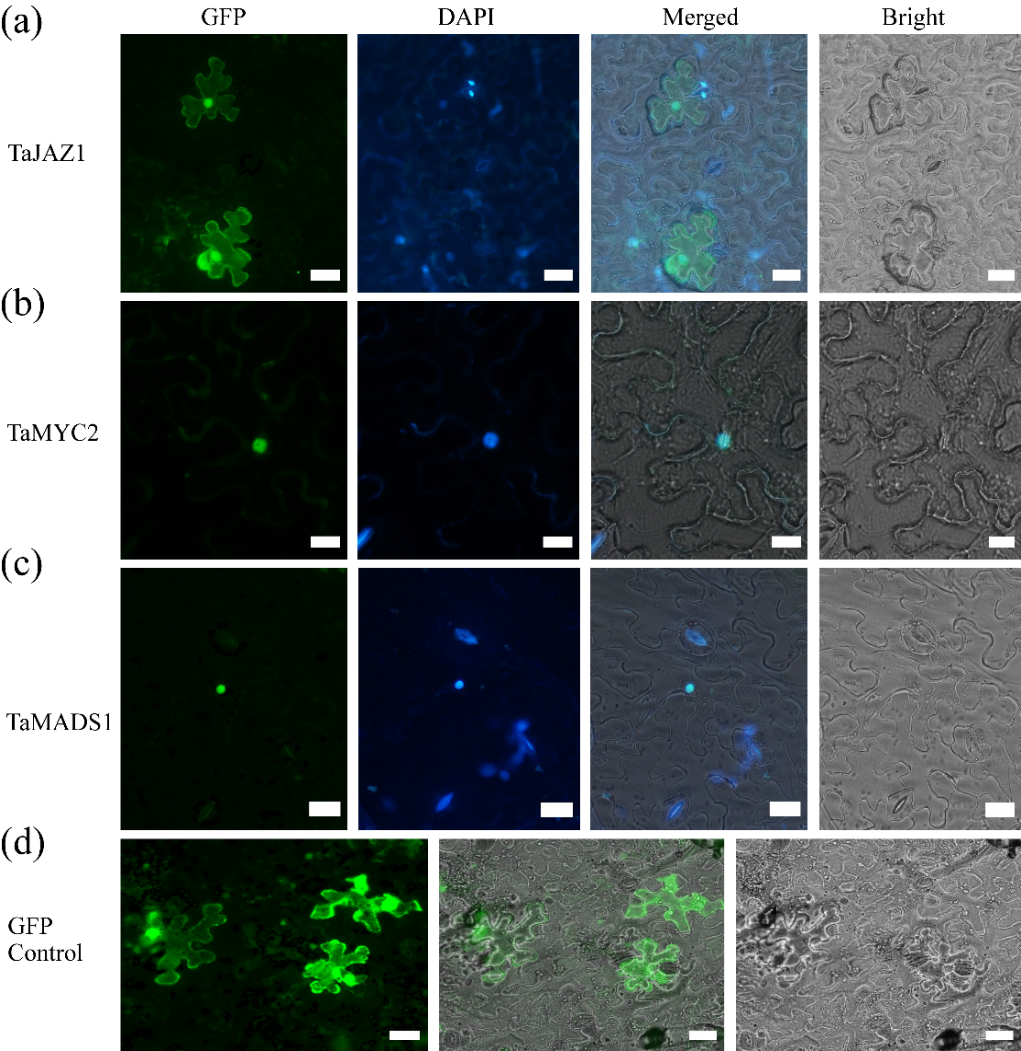


**Figure S12**. Subcellular localization of TaMYC2, TaJAZ1, and TaMADS1. TaJAZ1-GFP (a), TaMYC2-GFP (b), TaMADS1-GFP (c), and free GFP proteins (d) were transiently expressed in *N. benthamiana* leaves. Green fluorescence was visualized by fluorescence microscopy. Cellular integrity was assessed via bright-field imaging, and DAPI was used as a nuclear labeling marker. Scale bars: 100 μm.


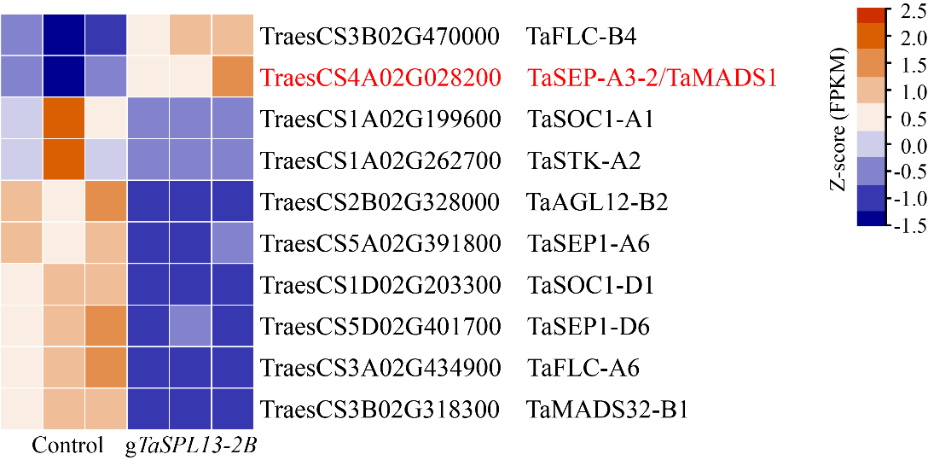


**Figure S13** *TaMADS1* is upregulated in the spikelets of *TaSPL13-2B* transgenic wheat. Heatmap of DEGs involved in floral organ determination. Gene expression values were calculated using Z-score (FPKM). Colors range from orange (high expression) to blue (low expression).

**
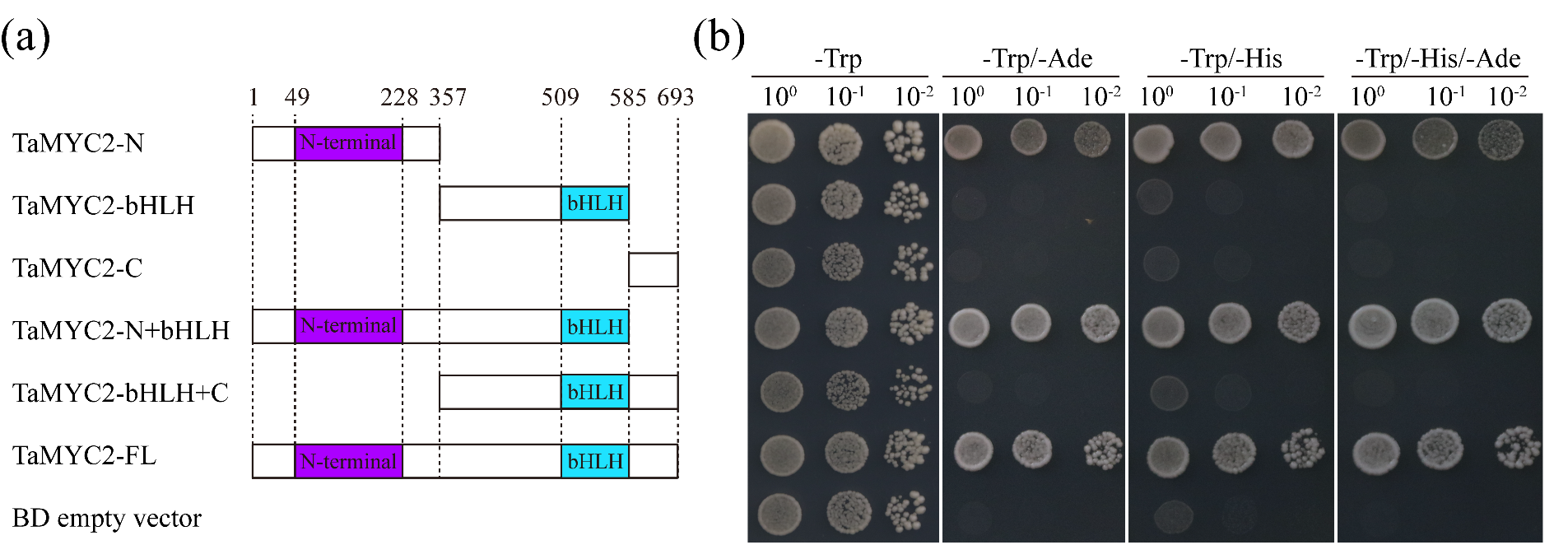
**

**Figure S14** Transcriptional activity analysis of the TaMYC2 protein. (a) Schematic diagram of full-length and truncated TaMYC2, highlighting its N-terminal region (purple) and bHLH domain (blue). (b) Serial dilutions of yeast transformants spotted onto SD/-Trp, SD/-Trp/-Ade, SD/-Trp/-His, and SD/-Trp/-His/-Ade media. Empty pGBKT7 vector served as the negative control.

**
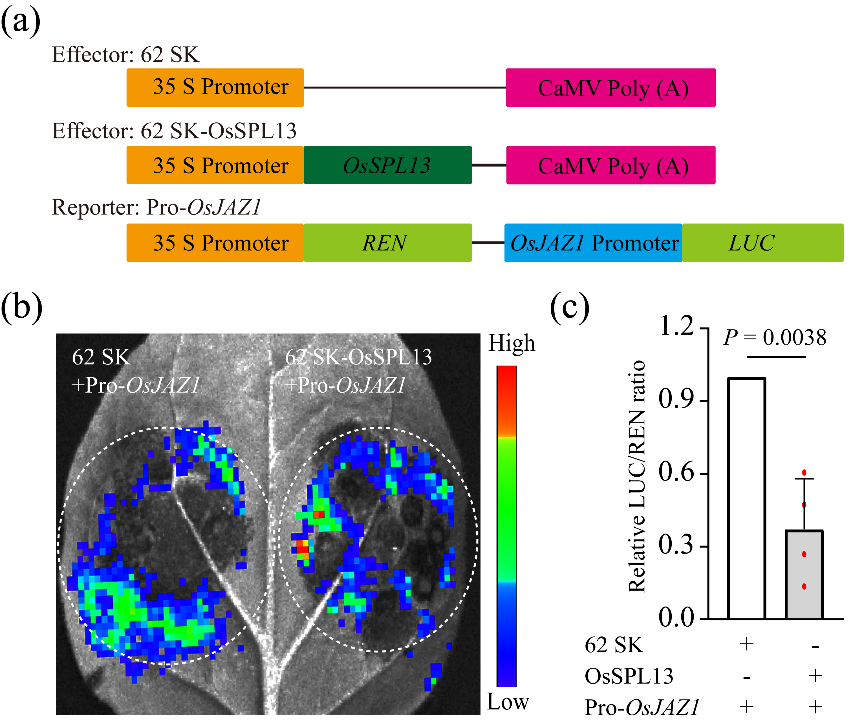
**

**Figure S15** OsSPL13 inhibits *OsJAZ1* expression. (a) Schematic of the reporter and effector constructs used for transient transactivation assays. (b) Representative transient expression image. (c) Luciferase (LUC) activity driven by the *OsJAZ1* promoter after coexpression with OsSPL13. REN served as the internal control; the LUC/REN ratio was used to asses *OsJAZ1* promoter activity. Two-tailed Student’s *t*-test was used to determine significant differences. Dots indicate the data distribution. Exact *P* values are shown in the figure.


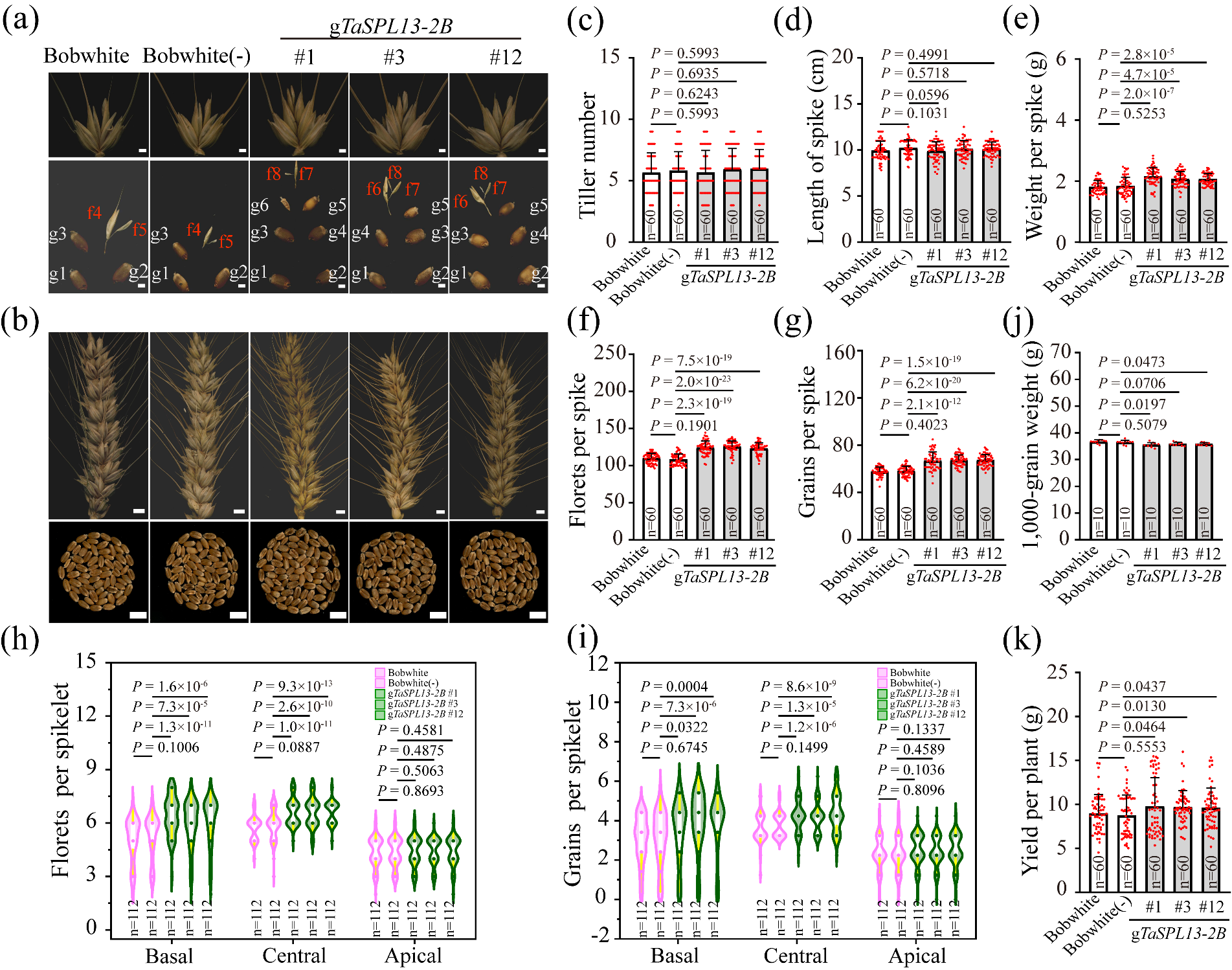


**Figure S16** Field performance of *TaSPL3-2B* T_8_ transgenic lines in Hongshan, Hubei Province, China from October 2021 to May 2022. Image showing the distribution of florets and grains on each spikelet (a) and grains per spike (b) between transgenic and control plants. Scale bars: 0.2 cm. (c-k) Statistical analysis of agronomic traits, including tiller number (c), spike length (d), weight per spike (e), florets per spike (f), grains per spike (g), florets per spikelet at basal, central and apical positions (h), grains per spikelet at these positions (i), thousand-grain weight (j), and yield per plant (k) between transgenic and control plants. g: grains; f: florets, red indicates aborted florets. Statistical differences were calculated using a two-tailed Student’s *t*-test. The four *P* values represent the significance differences between the null-segregant line and each of the three transgenic lines relative to wild type, and their exact *P* values are indicated in the figure. Dots show data distribution; n indicates the sample numbers.


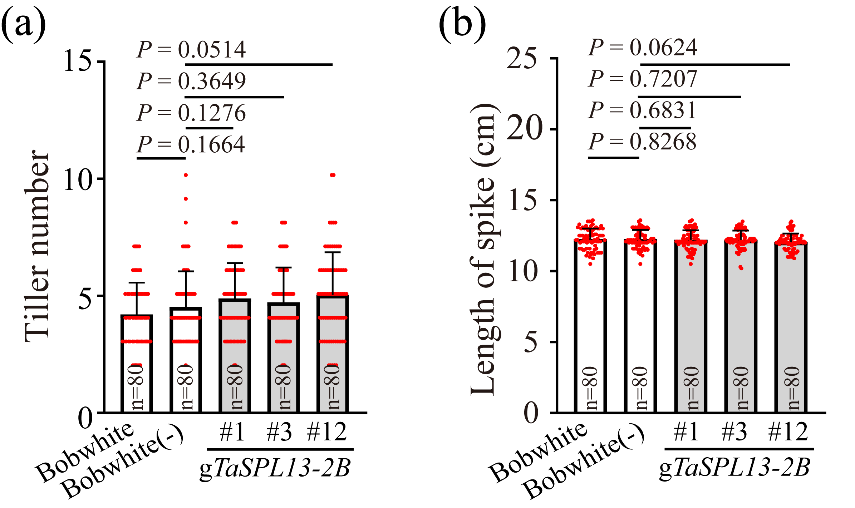


**Figure S17** Statistical comparison of *TaSPL13-2B* transgenic and control lines in the 2022/23 field season at the Hongshan experimental field. Comparisons of tiller number (a) and spike length (b) between control and three independent transgenic lines. Statistical differences were calculated using a two-tailed Student’s *t*-test. The four *P* values represent the significance differences between the null-segregant line and each of the three transgenic lines relative to wild type, and their exact *P* values are indicated in the figure. Dots show data distribution; n indicates the sample numbers.


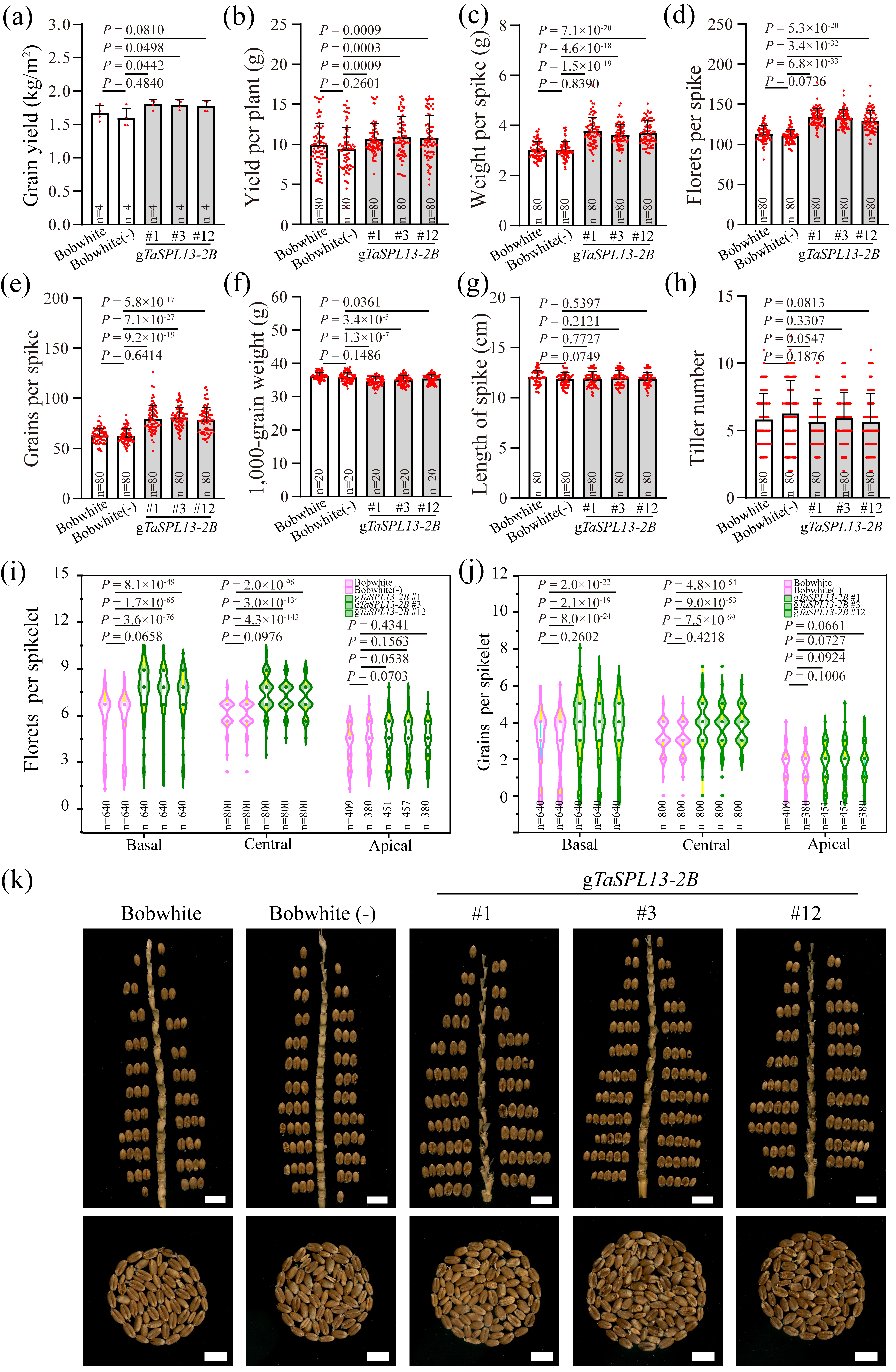


**Figure S18** Field experiments show improved yield traits in three *TaSPL13-2B* transgenic lines versus controls during the 2022/23 field season at the Xinzhou experimental field, with a randomized block design. (a-j) Statistical comparison of yield-related traits between *TaSPL13-2B* transgenic and control lines, covering grain yield (a), yield per plant (b), weight per spike (c), florets per spike (d), grains per spike (e), thousand-grain weight (f), spike length (g), tiller number (h), florets per spikelet (i), and grains per spikelet (j) at basal, central and apical positions. Statistical differences were calculated using a two-tailed Student’s *t*-test. The four *P* values represent the significance differences between the null-segregant line and each of the three transgenic lines relative to wild type, and their exact *P* values are indicated in the figure. Dots show data distribution; n indicates the sample numbers. (k) Morphology comparison of grains per spikelet along the spike, and grain numbers per spike between *TaSPL13-2B* transgenic and control lines. Scale bars: 0.7 cm.


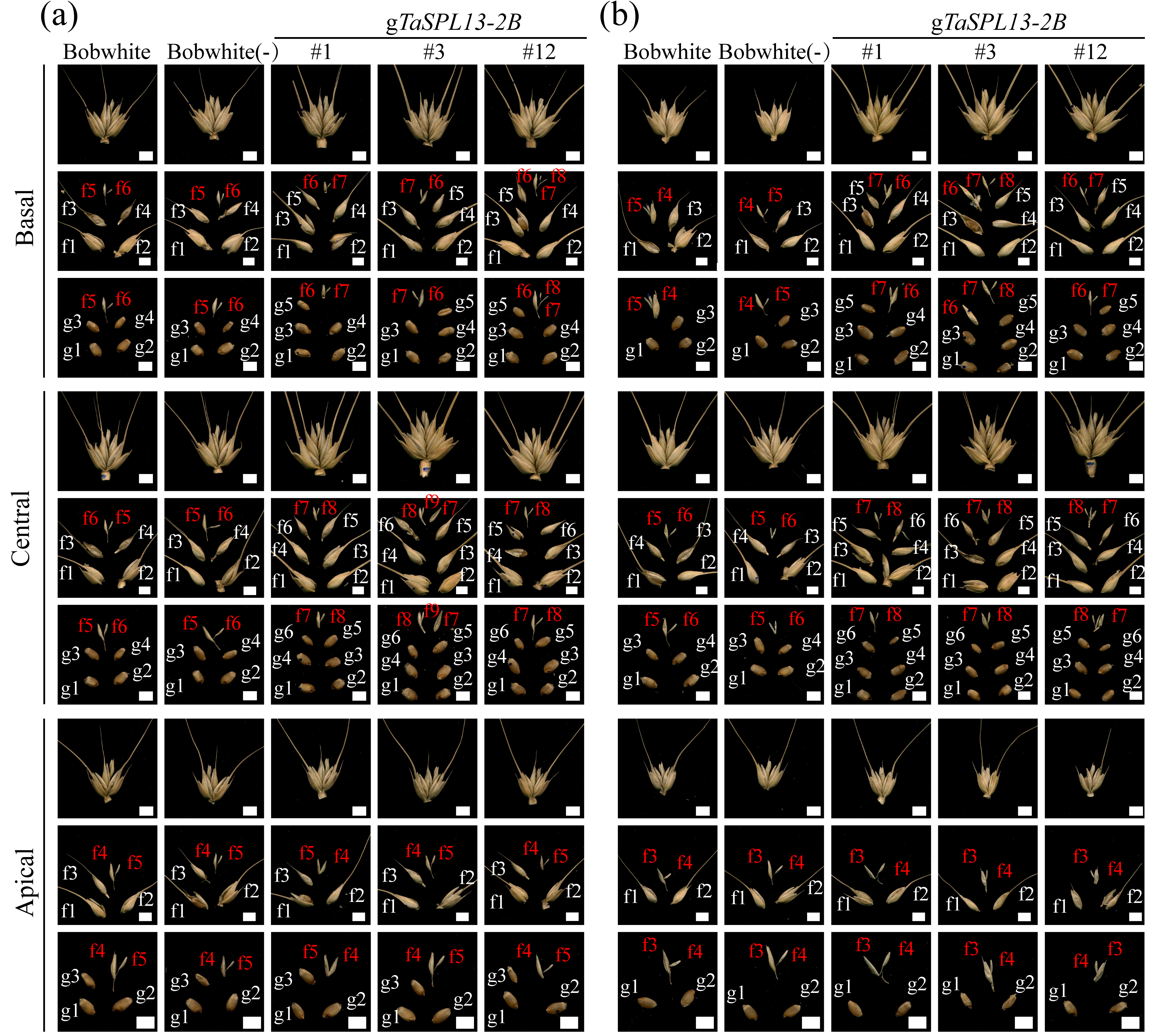


**Figure S19** Distribution of florets and grains per spikelet at apical, central, and basal positions on a single wheat spike. Comparison of florets and grains per spikelet at apical, central, and basal positions of wheat spikes in Hongshan (a) and Xinzhou (b). g: grain; f: floret; red represent abortive florets; white represent fertile florets. Scale bar: 0.3 cm.

**References**

Feng, N., Song, G.Y., Guan, J.T., Chen, K., Jia, M.L., Huang, D.H., Wu, J.J. *et al*. (2017) Transcriptome profiling of wheat inflorescence development from spikelet initiation to floral patterning identified stage-specific regulatory genes. *Plant Physiol.* **174**, 1779-1794.

Li, Y.P., Fu, X., Zhao, M.C., Zhang, W., Li, B., An, D.G., Li, J.M. *et al*. (2018) A genome-wide view of transcriptome dynamics during early spike development in bread wheat. *Sci. Rep.* **8**, 15338.

Lin, X.L., Xu, Y.X., Wang, D.Z., Yang, Y.M., Zhang, X.Y., Bie, X.M,. Gui, L.X. *et al*. (2024) Systematic identification of wheat spike developmental regulators by integrated multi-omics, transcriptional network, GWAS, and genetic analyses. *Mol. Plant* **17**, 438-459.

Pei, H.C., Teng, W., Gao, L.F., Gao, H.B., Ren, X.N., Liu, Y.H., Jia, J.Z. *et al*. (2023) Low-affinity SPL binding sites contribute to subgenome expression divergence in allohexaploid wheat. *Sci. China Life Sci.* **66**, 819-834.

Qi, P.F., Jiang, Y.F., Guo, Z.R., Chen, Q., Ouellet, T., Zong, L.J., Wei, Z.Z. *et al*. (2019) Transcriptional reference map of hormone responses in wheat spikes. *BMC Genom.* **20**, 390.
